# CD8^+^ T cells induce interferon-responsive oligodendrocytes and microglia in white matter aging

**DOI:** 10.1038/s41593-022-01183-6

**Published:** 2022-10-24

**Authors:** Tuğberk Kaya, Nicola Mattugini, Lu Liu, Hao Ji, Ludovico Cantuti-Castelvetri, Jianping Wu, Martina Schifferer, Janos Groh, Rudolf Martini, Simon Besson-Girard, Seiji Kaji, Arthur Liesz, Ozgun Gokce, Mikael Simons

**Affiliations:** 1 Institute for Stroke and Dementia Research, University Hospital of Munich, Ludwig Maximilian University (LMU) of Munich, Munich, Germany; 2 Institute of Neuronal Cell Biology, Technical University Munich, Munich, Germany; 3 German Center for Neurodegenerative Diseases, Munich, Germany; 4 Graduate School of Systemic Neurosciences, LMU Munich, Munich, Germany; 5 Munich Cluster of Systems Neurology, Munich, Germany; 6 Department of Neurology, Section of Developmental Neurobiology, University Hospital Würzburg, Würzburg, Germany

**Keywords:** Oligodendrocyte, Myelin biology and repair, Neural ageing, Neuroimmunology, Microglia

## Abstract

A hallmark of nervous system aging is a decline of white matter volume and function, but the underlying mechanisms leading to white matter pathology are unknown. In the present study, we found age-related alterations of oligodendrocyte cell state with a reduction in total oligodendrocyte density in aging murine white matter. Using single-cell RNA-sequencing, we identified interferon (IFN)-responsive oligodendrocytes, which localize in proximity to CD8^+^ T cells in aging white matter. Absence of functional lymphocytes decreased the number of IFN-responsive oligodendrocytes and rescued oligodendrocyte loss, whereas T-cell checkpoint inhibition worsened the aging response. In addition, we identified a subpopulation of lymphocyte-dependent, IFN-responsive microglia in the vicinity of the CD8^+^ T cells in aging white matter. In summary, we provide evidence that CD8^+^ T-cell-induced, IFN-responsive oligodendrocytes and microglia are important modifiers of white matter aging.

## Abstract (web summary)

Aging is associated with the formation of focal white matter lesions and atrophy. The authors discovered a role for CD8^+^ T cells in driving white matter-specific interferon responses in microglia and oligodendrocytes, leading to oligodendrocyte loss.

## Main

Age is the major risk factor for the most prevalent neurodegenerative diseases^1^. A better understanding of age-related alterations is therefore of overarching importance, but relatively little is known about the pathology occurring in the white matter, which is to a large extent composed of myelin, a lipid-rich membrane wrapped around axons by oligodendrocytes^2^. Myelination is not limited to early development but extends into adult life and contributes to brain plasticity. Regulated by neuronal stimuli and various environmental factors, there is a substantial fraction of adult-born oligodendrocytes that is actively engaged in forming new myelin sheaths, a process that declines in aging^3–5^. In humans, white matter volume starts to decline already in mid-life and these global alterations are often associated with focal lesions that appear hyperintense on magnetic resonance images^6–9^. Focal white matter degeneration is related to an increased risk of stroke and dementia^6^ and contributes to cognitive decline possibly by disrupting connective pathways in the brain^10–12^. In nonhuman primates and rodents, ultrastructural analyses of aging white matter show pathology of myelinated fibers, consisting of focal areas of degenerated myelin and axonal damage^13,14^. We have previously shown that such age-related myelin pathology results in a distinct white matter-associated microglia state, in which the disease-associated microglia (DAM) or microglia-neurodegenerative phenotype (MGnD) program is partially activated to clear myelin debris in groups of a few closely connected microglia^15–18^. Whereas the microglial responses to aging and disease are more intensely studied^15,17,19–21^, less is known about aging-related oligodendrocyte reactions related to myelin pathology. In the present study, we studied aging-induced glial reactivity and identified IFN-responsive oligodendrocyte and microglia in the white matter. We observed that CD8^+^ T cells increase in aging white matter and localize in close proximity to IFN-responsive cells. Genetic ablation of functional lymphocytes by using *Rag1*^−/−^ mice^22^ or *CD8*^−/−^ mice^23^ prevented aging-induced oligodendrocyte loss and IFN-responsive oligodendrocyte and microglia formation. Inversely, T-cell-checkpoint inhibition worsened the aging effect. These perturbation experiments support a role of CD8^+^ T cells in driving white matter aging.

## Results

### Transcriptomic aging responses of oligodendrocytes

**Fig. 1:**
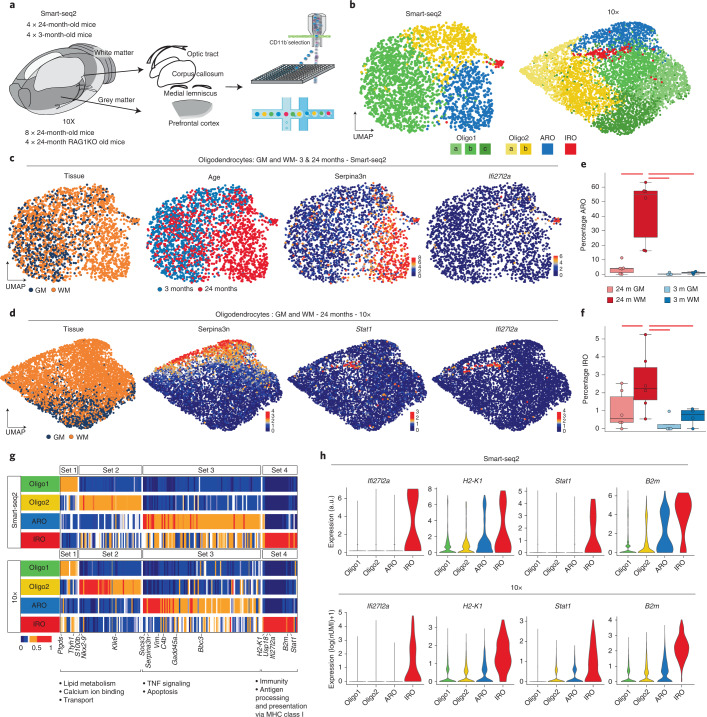
Identification of age-related gene expression signatures in oligodendrocytes. **a**, Experimental design from dissection to cell sorting and cell loading for the plate-based (Smart-seq2 (SS2)) and 10× pipelines, respectively. **b**, UMAP plots of oligodendrocytes in the SS2 and 10× datasets, colored by identified populations. **c**, UMAP plots of oligodendrocytes in the SS2 dataset, colored by tissue, age and expression of selected marker genes. **d**, The 10× dataset oligodendrocyte UMAP plots colored by tissue and expression of selected marker genes. **e**–**f**, Boxplots of the age-related oligodendrocytes (ARO) (**e**) and interferon-responsive oligodendrocytes (IRO) (**f**) cluster proportions per sample, respectively. The central line denotes the median, boxes represent the IQR and whiskers show the distribution except for outliers. Outliers are all points outside 1.5× the IQR. Each dot represents a sample (*n* = 20 independent experiments) and significant results (scCODA model) are indicated with red bars. 24 m, 24-month-old; 3 m, 3-month-old. **g**, Heatmaps of average expression of differentially expressed genes, comparing the four oligodendrocyte populations. Gene sets were identified as differentially expressed markers for each population using the SS2 dataset. Values are normalized per gene, showing the gene expression across populations. Each column represents a gene. GO terms are shown below each set of genes (Supplementary Table 3). TNF, tumor necrosis factor. **h**, Violin plots showing selected IRO-enriched marker genes across SS2 and 10× datasets. a.u., arbitrary units representing the corrected log(1*P*) (counts) value.

**Extended Data Fig. 1:**
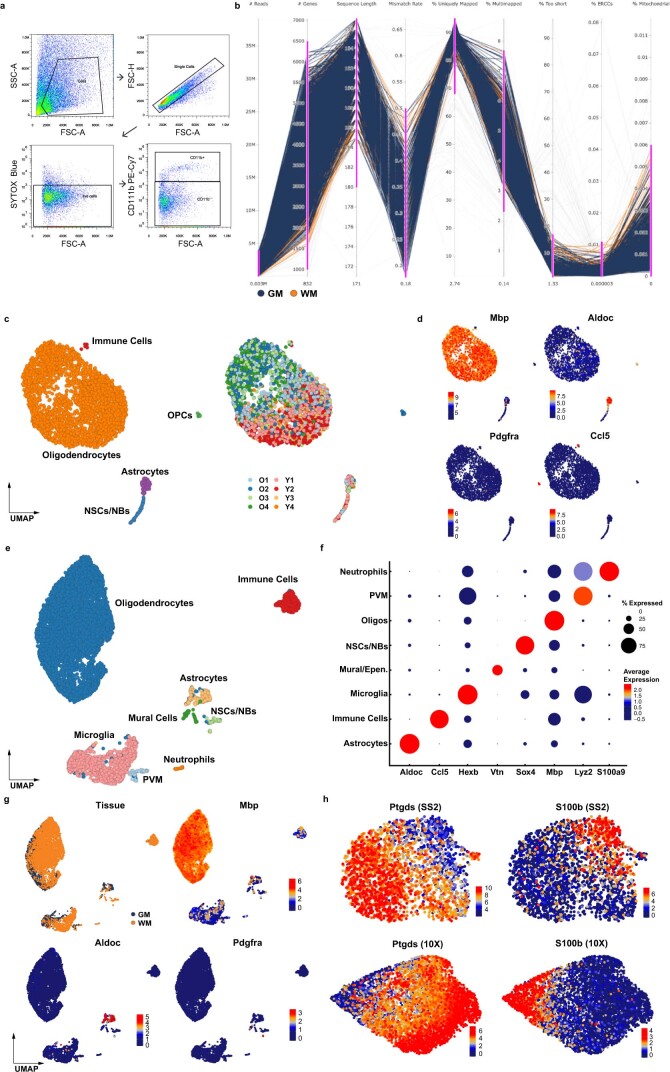
Sorting strategy and quality control of scRNA-seq data. **a**, Sorting strategy for the SS2 libraries. Flow cytometry gating of CD11b negative cells to enrich for oligodendrocytes and astrocytes. **b**, Parallel coordinates trace for Smart-seq2 dataset quality metrics. Distribution of 9 quantitative quality control metrics reported by fastqc or from early gene expression matrix analysis. Each line, continuously going through all metrics is a sample (single cell). Light grey samples were discarded based on windows of inclusion (depicted in fuchsia for each metric). Selected samples for the grey matter are in blue, white matter are in orange. From left to right, the metrics are: number of reads, number of genes, average length of sequence after trimming, mismatch rate during alignment, % of uniquely mapped reads, % of multimapped reads, % of reads considered too short, % of ERCCs, % of mitochondrial genes. 2538 single-cells passed the quality-control thresholds. **c**, UMAP plots of 2538 single-cell transcriptomes. Cell type clusters and samples from different animals are colour coded. O1-2-3-4 and Y1-2-3-4 correspond to samples from 24-month old and 3-month old animals, respectively. **d**, UMAP plots of selected cell type-specific marker genes in the SS2 dataset; *Mbp* (Oligodendrocyte), *Aldoc* (Astrocyte), *Pdgfra* (OPC), *Ccl5* (Immune cells). **e**, UMAP plot of 8726 single-cell transcriptomes in the 10x dataset. Different cell type clusters are color coded. **f**, Dot plot showing cell-type marker gene expression by clusters. **g**, UMAP plots of tissue annotation and cell-type specific marker genes in the 10x dataset. **h**, UMAP plots of the oligodendrocyte populations depicting the expression of Oligo1 and Oligo2 marker genes (*Ptgds* and *S100*b, respectively), in the SS2 and 10x dataset.

**Extended Data Fig. 2:**
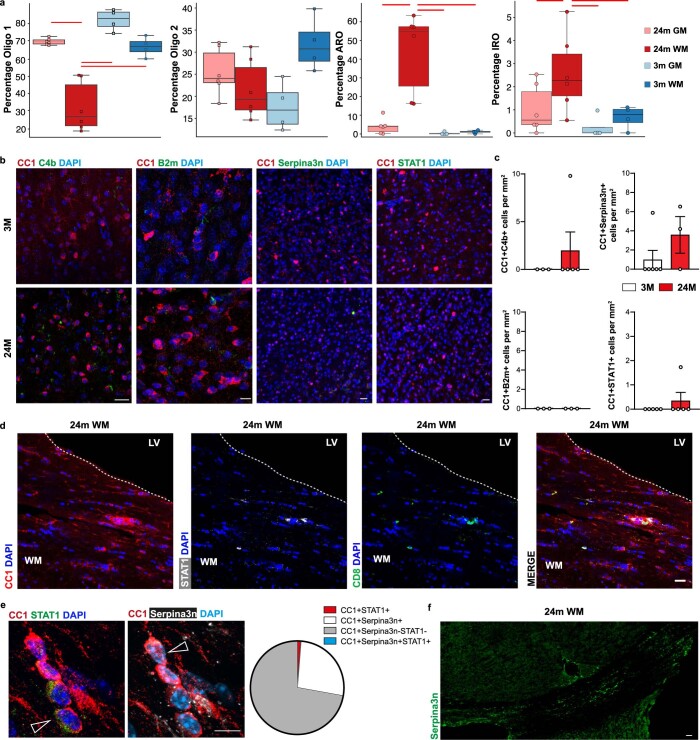
Characterization of age-related and interferon-responsive oligodendrocytes. **a**, Boxplots of the Oligo1, Oligo2, ARO and IRO cluster proportions per sample (n = 20 independent experiments). The central line denotes the median, boxes represent the interquartile range (IQR), and whiskers show the distribution except for outliers. Outliers are all points outside 1.5 times of the IQR. Each dot represents a sample and significant results (scCODA model) are indicated with red bars. **b**, Immunofluorescence showing expression of C4b, B2m, Serpina3n and STAT1 (green) in CC1^+^ oligodendrocytes (red) in the grey matter of 3- and 24- month old mice. Scale bar for C4b, Serpina3n and STAT1 20 µm, for B2m 10 µm. **c**, Quantification of CC1^+^ oligodendrocytes expressing C4b, B2m, Serpina3n and STAT1 at 3- and 24-month old mice in the grey matter (C4b^+^CC1^+^, 3 M, n = 3, 24 M, n = 5; Serpina3n^*+*^CC1^+^, 3 M, n = 6, 24 M, n = 3; B2m^+^CC1^+^, 3 M, n = 4, 24 M, n = 4; STAT1^+^CC1^+^, 3 M, n = 5, 24 M, n = 5; data are means±s.e.m.; *P* values from two-tailed Mann–Whitney test). **d**, Representative confocal images showing CD8^+^ T cells (green) and STAT1^+^CC1^+^ (white and red, respectively) oligodendrocytes in the white matter of 24-month old mice. **e**, Immunofluorescence showing expression Serpina3n (white) and STAT1 (green) in CC1^+^ oligodendrocytes (red) in 24-month old mice in the white matter. Scale bar, 10 µm. Arrows indicate cells positive for STAT1^+^CC1^+^ and Serpina3n^+^CC1^+^. Pie chart of percentage of different subpopulations. **f**, Representative confocal overview image of large showing Serpina3n distribution in the white matter of 24-month old mice. The experiment was repeated three times independently with similar results. Scale bar, 50 µm. Source data

To characterize the oligodendrocyte aging effect at single-cell resolution, we used two different single-cell RNA-sequencing (scRNA-seq) methods. For plate-based scRNA-seq (Smart-seq2), we dissociated gray matter from the frontal cortex and white matter tracts from the corpus callosum as well as the optical tracts, and the medial lemniscus from young (3-month-old) and aged (24-month-old), wild-type male mice (Fig. 1a). The scRNA-seq experimental details and animal information are reported according to guidelines^24,25^ (Methods and Supplementary Tables 1 and 2). To avoid isolation artifacts, we used our previously established automated dissociation protocol that inhibits ex vivo transcription^18^. We sorted live nonmyeloid (CD11b^−^ and SYTOX BLUE^−^) cells (Extended Data Fig. 1a). Each single-cell library passed through strict quality thresholds filtering out 112 single cells due to low quality and 2,538 single cells from 8 mice remained (Extended Data Fig. 1b). The cell-type composition of these cells was analyzed by unsupervised Uniform Manifold Approximation and Projection (UMAP) analysis (Fig. 1b, Extended Data Fig. 1c,d and Supplementary Table 1). Oligodendrocytes were separated into four different subclusters, of which the most abundant two clusters represent the heterogeneity of oligodendrocytes previously identified in juvenile and adult mouse by Marques et al.^26^. In our analysis, two additional oligodendrocyte clusters appeared in aged mice, which were enriched in white matter. One was characterized by a high expression of the serine (or cysteine) peptidase inhibitor, member 3N (Serpina3n) and the complement component C4b, previously associated with injury responses^27–32^. As this cluster was highly enriched in the aged white matter, we named it aging-related oligodendrocytes (Fig. 1b,c,e). We uncovered a smaller IFN-responsive oligodendrocyte subpopulation (IRO), which was characterized by the expression of genes commonly associated with an IFN response, such as *Stat1*, *Ifi27l2a* and major histocompatibility complex (MHC) class I-related genes (*H2-K1 and H2-D1)* (Fig. 1b,c,f). A related gene expression profile has been detected in oligodendrocyte progenitor cells (OPCs) in the context of multiple sclerosis^28,30^. To validate our results, we performed scRNA-seq using the 10× platform with cells from gray and white matter of 24-month-old mice (8,726 high-quality cells from 8 mice: Fig. 1b and Extended Data Fig. 1e). Tissues were prepared as described for Smart-seq2 and enriched for live cells using flow cytometry (Extended Data Fig. 1a). Major cell types were annotated based on canonical markers upon clustering (Extended Data Fig. 1e–g). We again identified a continuous range of oligodendrocytes that reproduced the major oligodendrocyte clusters of the Smart-seq2 scRNA-seq dataset (Fig. 1b,g). The higher number of oligodendrocytes allowed us to resolve Oligo1 and Oligo2 into five subclusters, but the identity and ratios of all four major clusters remained similar in both aged scRNA-seq datasets (Fig. 1g,h and Extended Data Fig. 1h). Using 20 independent scRNA-seq experiments, we compared changes in ratios of IROs and age-related oligodendrocytes by using the single-cell differential composition analysis (scCODA), which takes account of the compositionality of the scRNA-seq data and reliably controls for false discoveries^33^. Both age-related oligodendrocyte and IRO cluster proportions were significantly increased in the aged white matter samples (Fig. 1e,f). These increases were accompanied by a significant decrease in aged white matter Oligo1 but not in Oligo2 (Extended Data Fig. 2a).

### CD8^+^ T cells induce reactivity and loss of oligodendrocytes

**Fig. 2:**
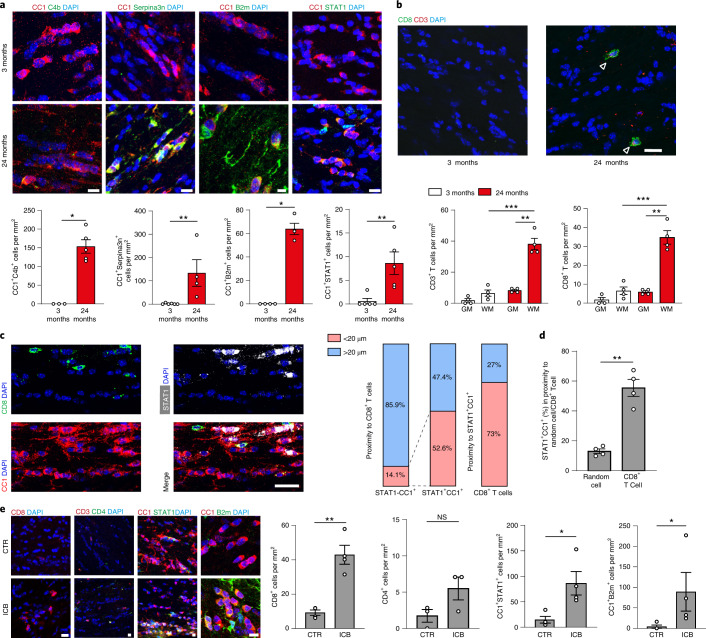
IFN-responsive oligodendrocytes localize to aged white matter close to CD8^+^ T cells. **a**, Immunofluorescence staining and quantification of C4b, Serpina3n, B2m and STAT1 in CC1^+^ oligodendrocytes in the white matter of 3- and 24-month-old mice (C4b^+^CC1^+^, 3-month, *n* = 3, 24-month, *n* = 5, ^*^P = 0.0357; Serpina3n^+^CC1^+^, 3-month, *n* = 6, 24-month, *n* = 4, ^**^P = 0.0095; B2m^+^CC1^+^, 3-month, *n* = 4, 24-month, *n* = 4, ^*^P = 0.0286; STAT1^+^CC1^+^, 3-month, *n* = 5, 24-month, *n* = 5, ^**^P = 0.0079; data are mean ± s.e.m.; *P* values are from a two-tailed Mann–Whitney *U*-test). Scale bar, 20 µm; for B2m, 10 µm. **b**, Immunofluorescence showing CD3^+^CD8^+^ T cells (indicated by arrowheads). Scale bar, 20 μm. Quantification of CD3^+^ T cells and CD8^+^ T cells in the gray (GM) and white (WM) matter of 3- and 24-month-old mice (*n* = 4 mice per group, 3-month WM versus 24-month WM, CD3^+^, ^***^P = 0.0003, CD8^+^, ^***^P = 0.0005; 24-month GM versus 24-month WM, CD3^+^, ^**^P = 0.0050, CD8^+^, ^**^P = 0.0032; data are mean ± s.e.m.; *P* values represent a two-sided Student’s *t*-test). **c**, Immunofluorescence of CD8^+^ T cells and STAT1^+^CC1^+^ oligodendrocytes in proximity in the white matter of 24-month-old mice. Scale bars, 20 μm. Bar plots show quantification of STAT1^+^ and STAT1^−^CC1^+^ proximity to CD8^+^ T cells and vice versa (3 sections per mouse were selected; a total of 134 CD8^+^ T cells and 272 STAT1^+^CC1^+^ oligodendrocytes from 4 mice were analyzed). **d**, Quantification of the percentage of STAT1^+^CC1^+^ oligodendrocytes found in proximity to random cells compared with CD8^+^ T cells (*n* = 4 mice per group, ^**^P = 0.0052; data are mean ± s.e.m; *P* value represents a two-sided paried Student’s *t*-test). **e**, Immunofluorescence staining and quantification of CD8^+^ T cells, CD4^+^ T cells, B2m^+^ and STAT1^+^CC1^+^ oligodendrocytes in the white matter of mice treated with anti-PD-1 and CTLA-4 (ICB) and isotype control antibodies (CTR) for 6 weeks starting at an age of 18 months (CD8^+^, *n* = 4, ^**^P = 0.0011; CD4^+^, *n* = 3; STAT1^+^CC1^+^, *n* = 4, ^*^P = 0.0244; B2m^+^CC1^+^, *n* = 4, ^*^P = 0.0286; data are mean ± s.e.m.; *P* values represent a two-sided Student’s *t*-test (CD8^+^, STAT1^+^CC1^+^) or two-tailed Mann–Whitney *U*-test (CD4^+^, B2m^+^CC1^+^)). Scale bar, 20 µm. NS, not significant. Source data

**Extended Data Fig. 3:**
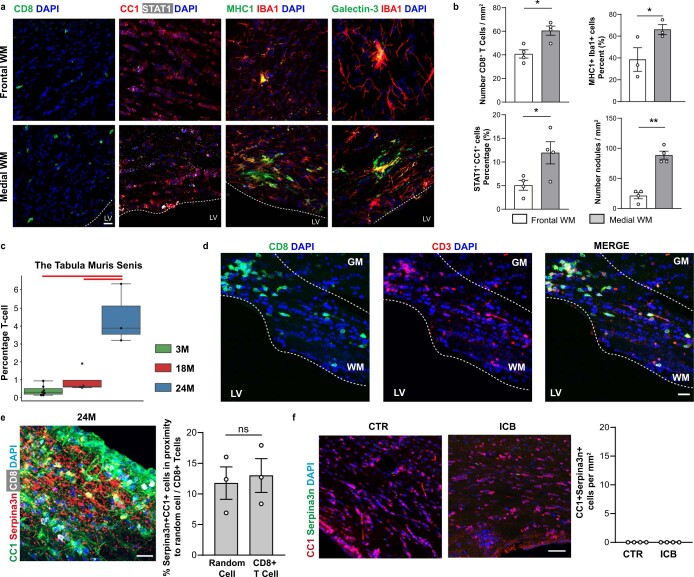
Characterization of CD8 + T cells in the aging white matter. **a**, Immunofluorescence staining showing the different densities of CD8^+^ T cells, STAT1^+^CC1^+^ cells, MHC1^+^IBA1^+^ cells and nodules (Galectin-3^+^Iba1^+^ cell clusters) between frontal white matter away from lateral ventricles and medial white matter close to lateral ventricles. Scale bar 20 µm. **b**, Quantification of CD8^+^ T cells, STAT1^+^CC1^+^ cells, MHC1^+^IBA1^+^ cells, and nodules (Galectin-3^+^IBA1^+^ cells cluster) density difference between frontal white matter and medial white matter of 24-month old mice (for CD8^+^, STAT1^+^CC1^+^, and nodules, n = 4 mice per group; CD8^+^, *P = 0.0438; STAT1^+^CC1^+^, *P = 0.0148; nodules, **P = 0.0088; for MHC1^+^IBA1^+^, n = 3 mice per group; *P = 0.0479; data are means±s.e.m. *P* values represent a two-sided paried Student’s t-test). **c**, T cells proportion per (n = 14 independent experiments) in the Tabula Muris Senis dataset. The central line denotes the median, boxes represent the interquartile range (IQR), and whiskers show the distribution except for outliers. Outliers are all points outside 1.5 times of the IQR. **d**, Representative confocal images of aged white matter stained for CD3 (red) and CD8 (green). The experiment was repeated three times independently with similar results. Scale bar, 20 µm. **e**, Immunofluorescence image showing CD8^+^ T cells (white) and Serpina3n^+^ (red) in CC1^+^ oligodendrocytes (green) in the white matter of 24-month old mice. Scale bars, 20 μm. Quantification of the percentage of Serpina3n^+^CC1^+^ oligodendrocytes found around random cells (<20 µm) compared to the percentage of Serpina3n^+^CC1^+^ oligodendrocytes found around CD8^+^ T cells (n = 3 mice per group; data are means±s.e.m.; *P* value represents a two-sided paried Student’s t-test). **f**, Immunofluorescence showing expression of Serpina3n (green) in CC1^+^ oligodendrocytes (red) in the white matter of mice treated with antibodies anti PD-1 and CTLA-4 and isotype control for 6 weeks starting at an age of 18 months. Scale bar 20 µm. Quantification of CC1^+^ oligodendrocytes expressing Serpina3n in the white matter of mice treated with antibodies anti PD-1 and CTLA-4 and isotype control antibodies (n = 4 mice per group; data are means±s.e.m). Source data

To validate the changes in scRNA-seq cluster ratios and to determine the localization of the age-related and the IFN-responsive oligodendrocytes, we costained anti-adenomatous polyposis coli (APC) clone CC1 (CC1^+^) oligodendrocytes by using antibodies against C4b, Serpina3n, B2M and STAT1. Consistent with the scRNA-seq data, we found that antibodies against C4b, Serpina3n, B2M and STAT1 labeled oligodendrocytes in the white matter, and only rarely in the gray matter (cortical areas of the brain) of aged (24-month-old) mice (Fig. 2a and Extended Data Fig. 2b,c). Colabeling against STAT1 and Serpina3n did not detect double-positive cells, in agreement with our scRNA-seq data that STAT1^+^ oligodendrocytes are distinct from Serpina3n^+^ oligodendrocytes (Extended Data Fig. 2e). Next, we compared the labeling in young (3-month) and old (24-month) gray and white matter and found that CC1^+^ oligodendrocytes, also immunoreactive for C4b, Serpina3n, STAT1 or B2M, are restricted to the aged brains mostly in the white matter (Fig. 2a and Extended Data Fig. 2b,c). Quantification revealed that about 3–5% of the CC1^+^ cells within the corpus callosum of 24-month-old mice were positive for the markers STAT1 and B2M. We found that C4b^+^/Serpina3n^+^ oligodendrocytes were more abundant (41% of CC1^+^ cells were Serpina3n^+^/CC1^+^ and 30% C4b^+^/CC1^+^ in 24-month-old white matter) and also more evenly distributed compared with B2M^+^/STAT1^+^ oligodendrocytes (Fig. 2a and Extended Data Fig. 2d,f). Our subregional localization analysis revealed that STAT1^+^ oligodendrocytes were localized significantly closer to the medial white matter bordering the lateral ventricles compared with the frontal white matter (Extended Data Fig. 3a,b). Previous work has identified infiltrating T cells in the aged brain, close to neurogenic niches and within the optic nerve^34,35^. As T cells are a major source of IFNs, we analyzed T-cell proportions in the mouse aging single-cell transcriptomic atlas^35^ and found that the T cells significantly increased in 24-month-old mice compared with 18- and 3-month-old mice (Extended Data Fig. 3f). Using immunohistochemistry, we analyzed the CD3^+^ T cells in the white and gray matter of the aged brain and observed that the T cells, which were mostly CD8^+^ T cells, were almost exclusively found in the white matter, where they were enriched in areas close to the lateral ventricles (Fig. 2b and Extended Data Figs. 2d and 3a,b,d). Next, we studied the spatial relationship between the IFN-responsive oligodendrocytes (STAT1^+^CC1^+^) and CD8^+^ T cells in aging white matter. Strikingly, STAT1^+^CC1^+^ cells are more frequently localized in close proximity to CD8^+^ T and vice versa (<20 µm) (Fig. 2c). Moreover, STAT1^+^CC1^+^ oligodendrocytes are found significantly more often in close proximity to CD8^+^ T cells than randomly chosen DAPI^+^ cells (Fig. 2d), which was not the case for Serpin3n^+^CC1^+^ oligodendrocytes (Extended Data Fig. 3e).

**Extended Data Fig. 4:**
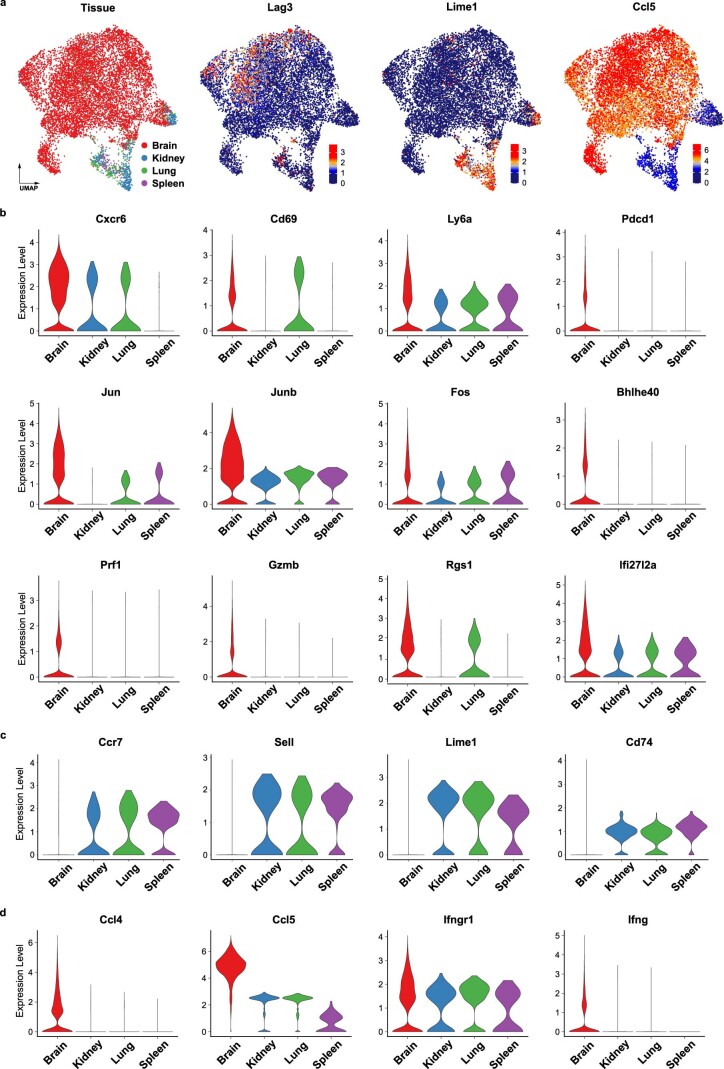
Comparison of transcriptome prolife of CD8 + T cells within the brain compared to other organs. **a**, UMAP plots of 9732 CD8^+^ T cells isolated from published aged mouse scRNA-seq datasets; Brain CD8^+^ T cells (3 independent experiments taken from the Groh et al^34^ and the Tabula Muris Senis datasets^36^), Kidney, Lung and spleen CD8^+^ T cells (2-4 independent experiments taken from the Tabula Muris Senis 21- and 24-months old datasets), colored by tissue annotation and the expression of selected marker genes. **b**^**−**^**c**, Violin plots of up-regulated (b) and down-regulated (c) genes in the brain compared to kidney, lung and spleen. **d**, Violin plots of differentially expressed cytokine and cytokine receptor genes in the brain.

To characterize the CD8^+^ T cells in the aging brain, we analyzed the mouse aging single-cell transcriptomic atlas^36^ and the CD8^+^ T-cell dataset from Groh et al.^34^. Our analysis, which included samples from 4 different organs of 21- and 24-month-old mice, showed that brain-associated CD8^+^ T cells segregated away from CD8^+^ T cells from the spleen, kidney and lung in the UMAP presentation (Extended Data Fig. 4a). Genes differentially upregulated in the brain CD8^+^ T cells were enriched in tissue-resident memory T-cell markers (*Cxcr6*, *Cd69*, *Junb*, *Bhlhe40*), checkpoint molecules (*Pdcd1*) and effector function-associated genes (*Gzmb*, *Ccl4*, *Ccl5*, *Ifng*), but low in genes associated with central memory T cells (*Sell*, *Ccr7*) compared with CD8^+^ T cells from the spleen, kidney and lung (Extended Data Fig. 4a–d).

**Extended Data Fig. 5:**
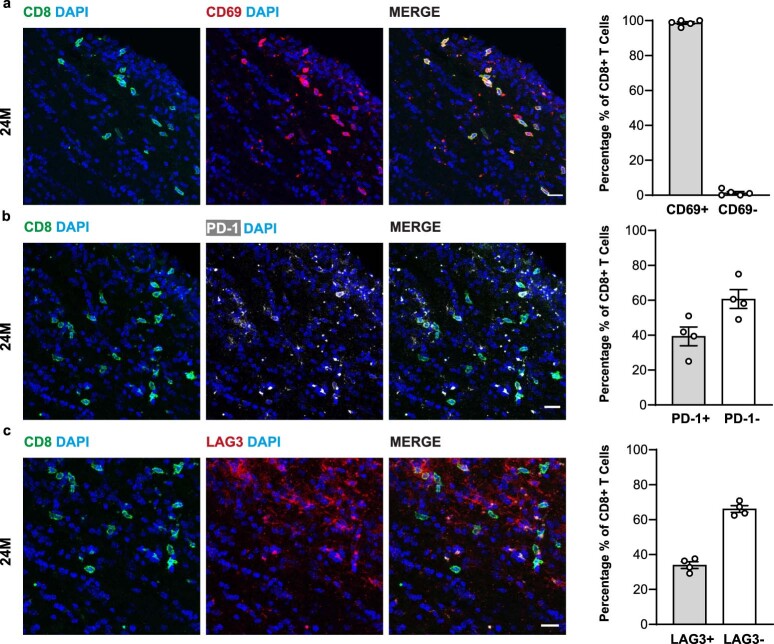
CD8 + T cells within the aged white matter express tissue-resident memory and checkpoint markers. **a**, Representative confocal images of aged white matter stained for CD8 (green) with CD69 (red). Scale bar, 20 µm. Quantification of the percentage of CD8^+^ and CD69^+^ T cells in the aged white matter (three sections per mouse; 256 CD8^+^ T cells were analyzed from 4 mice; data are means±s.e.m). **b**, Representative confocal images of aged white matter stained for CD8 (green) with PD-1 (white). Scale bar, 20 µm. Quantification of the percentage of CD8^+^ and PD-1^+^ cells in the aged white matter (three sections per mouse; 237 CD8^+^ T cells were analyzed from 4 mice; data are means±s.e.m). **c**, Representative confocal images of aged white matter stained for CD8 (green) with LAG3 (red). Scale bar, 20 µm. Quantification of the percentage of CD8^+^ and LAG3^+^ cells in the aged white matter (three sections per mouse; 237 CD8^+^ T cells were analyzed from 4 mice; data are means±s.e.m). Source data

Next, we performed immunofluorescence staining for the tissue-resident memory T-cell marker, CD69, which showed that almost all the CD8^+^ T cells were also positive for CD69 (Extended Data Fig. 5a). We analyzed the expression of the checkpoint molecules, programmed cell death protein 1 (PD-1) and the lymphocyte-activation gene 3 (*LAG-3*) and found that 40% of CD8^+^ T cells were immunolabeled by antibodies against PD-1 and 32% of CD8^+^ T cells by antibodies against *LAG-3* (Extended Data Fig. 5b,c).

**Extended Data Fig. 6:**
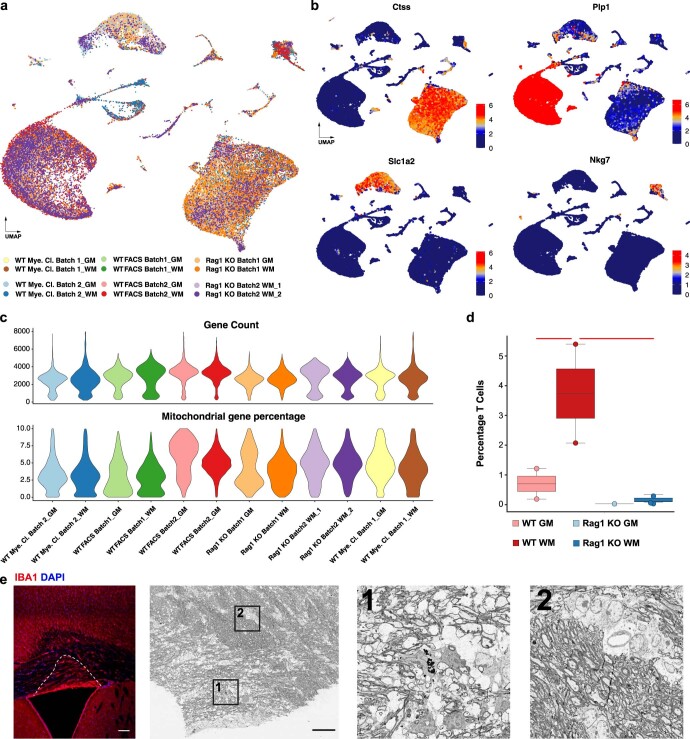
T cell characterization and evidence of focal areas of hypomyelination in the aged white matter. **a**, UMAP plot of 44938 single-cell transcriptomes. Sequencing runs are color-coded. **b**, UMAP plots selected cell-type specific marker genes. **c**, Violin plots of gene count and mitochondrial gene percentage per cell in each sample. **d**, Boxplot of the T cells (Fig. 3a,b) proportion per sample (n = 8 independent experiments), respectively. The central line denotes the median, boxes represent the interquartile range (IQR), and whiskers show the distribution except for outliers. Outliers are all points outside 1.5 times of the IQR. Each dot represents a sample and significant results (scCODA model) are indicated with red bars. **e**, Confocal microscopy image of a vibratome brain section from 24-month old wild type mice stained for Iba1 (red) and DAPI (blue). Scale bar 100 µm. Correlative Light and Electron Microscopy of the corpus callosum in the aged mice with two boxed regions labeled as 1 and 2. Scanning electron micrograph of the same region on the adjacent vibratome section showing normal myelination next to areas with hypomyelination. Scale bar 50 µm. Boxed regions of thinner myelin with myelin fragments (1) and normal myelin (2) are shown at higher resolution. The experiment was repeated three times independently with similar results.

As checkpoint molecules are known to control the function of T cells, we treated 18-month-old mice with antibodies against the coinhibitory receptors such as cytotoxic T-lymphocyte-associated protein 4 (CTLA-4) and PD-1 to determine the effect on oligodendrocytes. Immune checkpoint blockage therapy is used to dampen coinhibitory molecules to achieve robust anti-tumor immune response^37^. Treatment of mice with twice-weekly intraperitoneal injections with anti-PD-1 and anti-CTLA-4 antibodies for 6 weeks resulted in an increased number of CD8^+^ T cells, but not CD4^+^ T cells, in the white matter (Fig. 2e). In addition, the number of STAT1^+^CC1^+^ and B2M^+^CC1^+^ oligodendrocytes increased within the corpus callosum by checkpoint blockage therapy (Fig. 2e). The formation of Serpina3n^+^ oligodendrocytes was not induced by anti-PD-1/anti-CTLA-4 antibody treatment (Extended Data Fig. 6c).

**Fig. 3:**
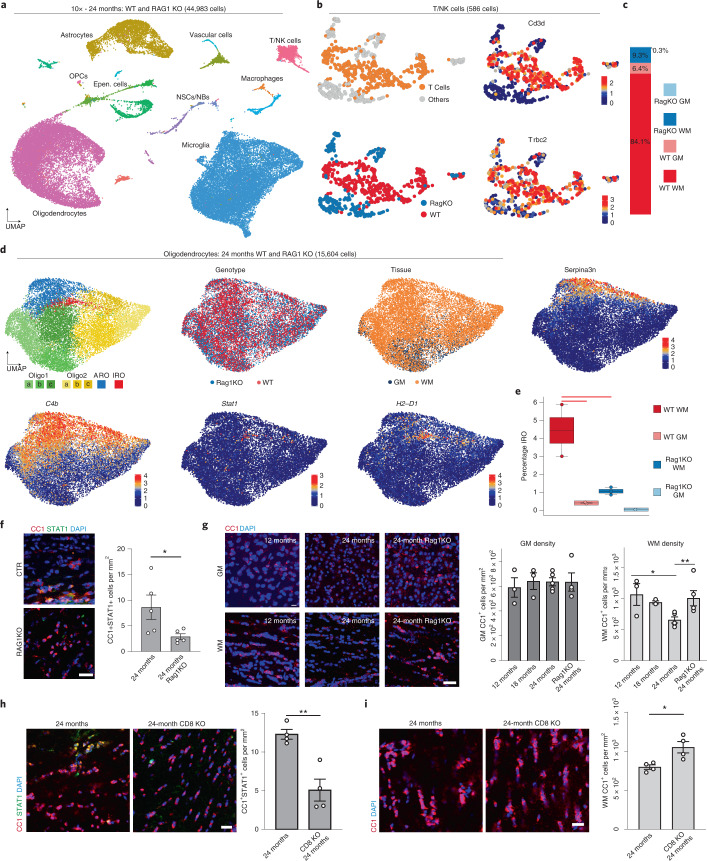
Absence of functional lymphocytes reduces IFN-responsive oligodendrocyte numbers and increases oligodendrocyte cell density in the aged white matter. **a**, UMAP plot of 44,983 single-cell transcriptomes, colored by major cell types. **b**, UMAP of T cells and NK cells, colored by T-cell identity, genotype and T-cell marker genes. **c**, Bar plot showing the relative distribution of each genotype-tissue experimental group within the T/NK cell population. **d**, UMAP plots of oligodendrocytes in the *Rag1*^−/−^ and wild-type integrated dataset, colored by identified clusters, genotype and tissue annotation, as well as selected marker genes. **e**, Boxplot of the IRO cluster proportion per sample. The central line denotes the median, boxes represent the IQR and whiskers show the distribution except for outliers. Outliers are all points outside 1.5× the IQR. Each dot represents a sample (*n* = 8 independent experiments) and significant results (scCODA model) are indicated with red bars. **f**, Immunofluorescence staining and quantification of STAT1^+^CC1^+^ oligodendrocytes in the white matter of 24-month-old wild-type and 24-month-old *Rag1*^−/−^ mice (*n* = 5 mice per group, ^*^P = 0.0466; data are mean ± s.e.m.; *P* values represent a two-sided Student’s *t*-test). Scale bar, 20 µm. **g**, Immunofluorescence staining and quantification of CC1^+^ oligodendrocyte density in the gray (GM) and white matter (WM) of 12-, 18- and 24-month-old wild-type and *Rag1*^−/−^ mice (GM, *n* = 3, 3, 5, 4; WM, *n* = 3, 3, 5, 4; 12 months versus 24 months, ^*^P = 0.0185, 24-month versus 24-month *Rag1*^−/−^, ^**^P = 0.0083; data are mean ± s.e.m.; *P* values represent two-sided, one-way ANOVA with post hoc Tukey’s test). Scale bar, 20 µm. **h**, Immunofluorescence staining and quantification of CC1^+^STAT1^+^ oligodendrocytes in the white matter of 24-month-old wild-type and *CD8*^−*/*−^ mice (*n* = 4 mice per group, ^**^P = 0.0035; data are mean ± s.e.m.; *P* value represents a two-sided Student’s *t*-test). Scale bar, 20 µm. **i**, Immunofluorescence staining and quantification of CC1^+^ oligodendrocyte density (red) in the white matter of 24-month-old wild-type and CD8^−/−^ mice (*n* = 4 mice per group, ^*^P = 0.0176; data are mean ± s.e.m.; *P* value represents a two-sided Student’s *t*-test). Scale bar, 20 µm. Source data

To continue exploring the link between T cells and white matter aging, we performed three independent scRNA-seq experiments using the 10× platform on 24-month-old *Rag1*^−/−^ mice, which lack functional lymphocytes. The *Rag1*^−/−^ dataset integrated well with our 24-month-old wild-type 10× datasets, showing good batch mixing while still preserving biological variance (Fig. 3a and Extended Data Fig. 6a–c). The unsupervised clustering of T/natural killer (NK) cells showed that the number of *Cd3d*- and *Trbc2*-expressing T cells are significantly higher in the white matter of aged wild-type mice (Fig. 3b,c and Extended Data Fig. 6d). Unsupervised clustering of oligodendrocytes from the combined datasets showed again the same transcriptional clusters with *Stat1* and *H2-D1* expressing IROs and Serpina3n expressing age-related oligodendrocytes (Fig. 3d). The scCODA analysis showed a marked reduction of IROs in *Rag1*^−/−^ mice (Fig. 3e). This finding was consistent with our immunolabeling experiments, which showed a reduction of IROs in *Rag1*^−/−^ mice (Fig. 3f). Together, these data provide evidence that the adaptive immune system promotes IFN responses in oligodendrocytes in the aging white matter, but to what extent these changes contribute to white matter degeneration is unclear.

**Extended Data Fig. 7:**
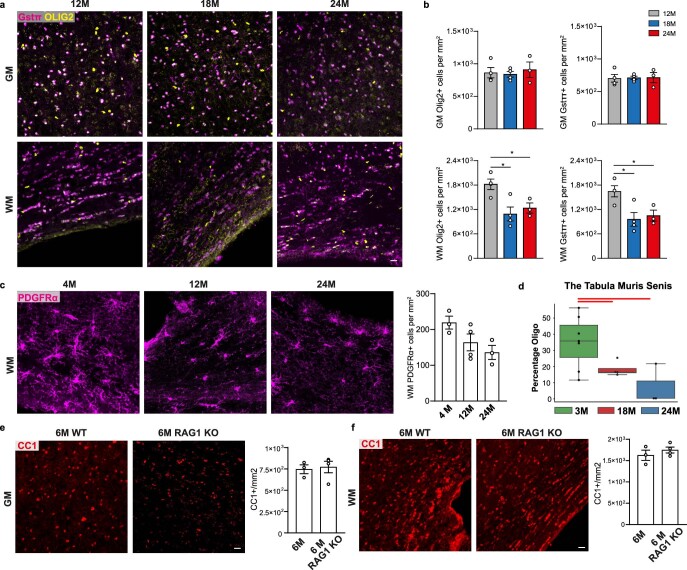
Evidence of oligodendrocyte loss in the aged white matter. **a**, Immunofluorescence showing OLIG2^+^ oligodendrocytes lineage cells (yellow) and oligodendrocytes GSTπ^+^ (magenta) and in the grey and white matter of 12-, 18-, 24-month old mice. Scale bar 20 µm. **b**, Quantification OLIG2^+^ and GSTπ^+^ cells in the grey and white matter of 12-, 18-, 24-month old mice (for all the graphs in b, n = 4,4,3 mice per group; Olig2^+^ cells in WM, 12 M versus 24 M, *P = 0.0493, 12 M versus 18 M, *P = 0.0123; GSTπ^+^ cells in WM, 12 M versus 24 M, *P = 0.0481, 12 M versus 18 M, *P = 0.0180; data are mean±s.e.m.; *P* values represent two sided one-way ANOVA with post hoc Tukey test). **c**, Immunofluorescence showing and PDGFRα^+^ cells (magenta) in the white matter of 4-, 12-, 24-month old mice. Scale bar 20 µm. Quantification PDGFRα^+^ OPCs (magenta) in the white matter of 4-, 12-, 24-month old mice (PDGFRα^+^, n = 3,4,3; data are mean ± s.e.m.; *P* value represents two sided, one-way ANOVA with post hoc Tukey test)**. d**, Oligodendrocytes proportion per sample (n = 14 independent experiments) in the Tabula Muris Senis dataset. The central line denotes the median, boxes represent the interquartile range (IQR), and whiskers show the distribution except for outliers. Outliers are all points outside 1.5 times of the IQR. **e-f**, Immunofluorescence showing CC1^+^ oligodendrocytes (red) in the grey matter and white matter of 6-month old wild type and *Rag1*^−/−^ mice. Scale bar, 20 µm. Quantification of CC1^+^ oligodendrocytes in the grey matter and white matter of 6-month old wild type and *Rag1*^−/−^ mice (n = 3,4 mice per group, mean ± s.e.m.). Source data

Next, we used correlated light and electron microscopy to detect areas of high IBA1^+^ cell density by immunohistochemistry, followed by scanning electron microscopy to determine the ultrastructure of myelinated axons. This analysis uncovered focal areas of hypomyelination in the corpus callosum of 24-month-old mice close to the ventricular area (Extended Data Fig. 6e). To quantify the age-related decay, we determined the number of oligodendrocytes in the aging brain. First, we analyzed oligodendrocyte proportions by using the mouse aging single-cell transcriptomic atlas^36^ and found that the oligodendrocyte significantly decreased in 24-month-old compared with 18- and 3-month-old mice (Extended Data Fig. 7d). Next, we used immunohistochemistry to quantify oligodendrocyte cell numbers in 12-, 18- and 24-month-old mice (Fig. 3g). We observed that the density of CC1^+^ oligodendrocytes declined in the 24-month-old compared with the 12-month-old white matter, whereas no changes were observed in the aged gray matter (Fig. 3g). Similar results were obtained when glutathione *S*-transferase (GST-π) was used as an additional marker to stain for mature oligodendrocytes (Extended Data Fig. 7a,b). In addition, there was a decrease in Olig2^+^ oligodendroglial cells, but not of Pdgfra^+^ OPCs (Extended Data Fig. 7c). Next, we analyzed oligodendrocytes in *Rag1*^−/−^ mice and found no differences in oligodendrocyte density compared with controls at 6 months in both gray and white matter (Extended Data Fig. 7e,f). However, when 24-month-old mice were analyzed, we detected a higher density of oligodendrocytes in the white matter of 24-month-old *Rag1*^−/−^ compared with wild-type control mice (Fig. 3g).

As *Rag1*^−/-^ mice lack various populations of mature adaptive immune cells, including CD4, CD8, gamma–delta and B cells, we used mice homozygous for the Cd8a^tm1Mak^-targeted mutation (CD8^−/−^) that specifically lacks functional CD8^+^ T cells^23^. When we analyzed 24-month-old CD8^−/−^ mice, we found a reduction of the number of STAT1^+^CC1^+^ oligodendrocytes compared with control wild-type mice (Fig. 3h). Next, we determined the density of CC1^+^ oligodendrocytes and detected higher numbers of oligodendrocytes in 24-month-old CD8^−/−^ mice compared with controls (Fig. 3i), confirming that CD8^+^ T cells induce IFN responses and loss of oligodendrocytes.

### CD8^+^ T cells induce IFN-responsive microglia

**Fig. 4:**
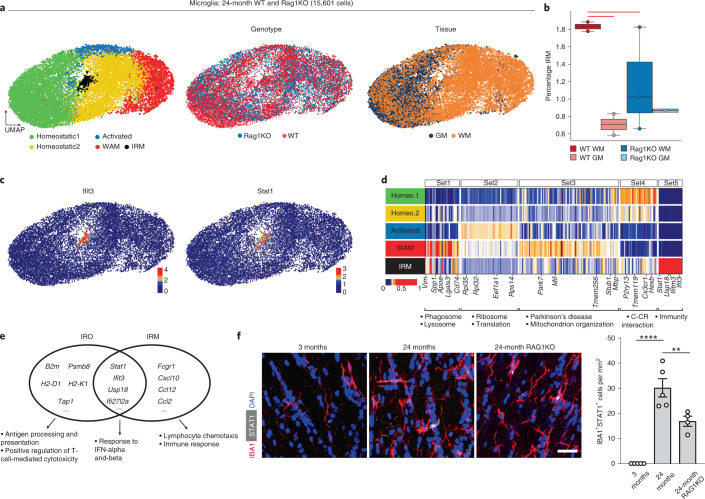
Identification of IFN-responsive microglia in aged white matter. **a**, UMAP plots of microglia colored by identified clusters, genotype and tissue annotation. **b**, Boxplot of the IRM cluster proportion per sample, respectively. WAM, White matter associated microglia; WT, Wild-type. The central line denotes the median, boxes represent the IQR and whiskers show the distribution except for outliers. Outliers are all points outside 1.5× the IQR. Each dot represents a sample (*n* = 8 independent experiments) and significant results (scCODA model) are indicated with red bars. **c**, UMAP plots of selected IRM marker genes. **d**, Heatmaps of average expression of differentially expressed genes, comparing the five microglia populations. Gene sets 1–4 were identified in Safaiyan et al.^18^ and set 5 was identified by differential expression analysis of the IRM cluster. Values are normalized per gene, showing the gene expression across populations. Each column represents a gene. GO terms are shown below each set of genes. **e**, Venn diagram of top 50 differentially expressed genes of IRO and IRM clusters with an intersection set of 16 genes. Gene lists are found in Supplementary Table 3. **f**, Immunofluorescence staining and quantification of STAT1^+^IBA1^+^ microglia in the white matter of 3- and 24-month-old wild-type and 24-month-old *Rag1*^−/−^ mice (IBA1^+^STAT1^+^, *n* = 5,5,4; 3 months versus 24 months, ^***^P = 0.000002: 24-month versus 24-month *Rag1*^−/−^, ^**^P = 0.0087; data are mean ± s.e.m.; *P* value represents two-sided, one-way ANOVA with post hoc Tukey’s test). Scale bar, 20 µm. Source data

As previous studies have identified IFN-responsive microglia in various models of neurodegenerative disease and during aging^15^, we asked whether the adaptive immune system mediates IFN-responsive microglia conversion. Unsupervised clustering of 15,601 microglia from the aged white and gray matter of *Rag1*^−/−^ and wild-type mice revealed distinct populations, including previously described homeostatic microglia, activated microglia, white matter-associated microglia and, in addition, a smaller population of IFN-responsive microglia (Fig. 4a). The IFN-responsive microglia subset was significantly enriched in aged white compared with gray matter and was reduced in aged *Rag1*^−/−^ mice (Fig. 4a,b). INF-responsive microglia are characterized by the upregulation of IFN-stimulated genes *Stat1* and *Ifit3* (Fig. 4c,d). Gene ontology (GO) enrichment analysis showed that IFN-responsive microglia and oligodendrocytes share a transcriptional signature of IFN-induced genes including *Stat1*, *Ifit3*, *Usp18* and *Ifit27l2a* (Fig. 4e). GO enrichment analysis also detected differences with upregulated genes involved in antigen processing and positive regulation of T-cell-mediated cytotoxicity for IFN-responsive oligodendrocytes, lymphocyte chemotaxis and immune responses for IFN-responsive microglia.

**Fig. 5:**
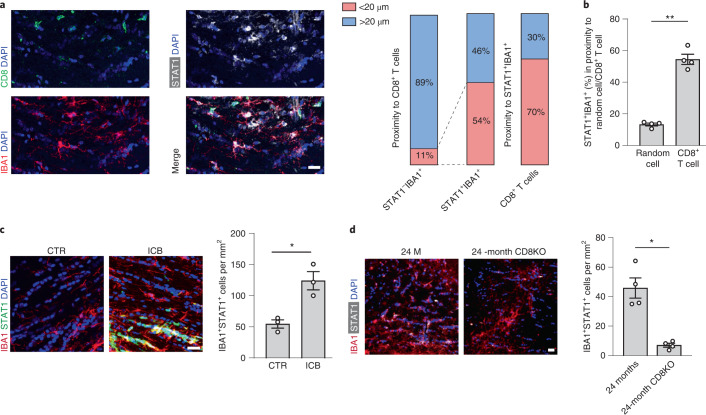
CD8^+^ T cells induce an IFN-responsive microglia state in the aged white matter. **a**, Immunofluorescence staining and quantification of CD8^+^ T cells (green) and STAT1^+^IBA1^+^ microglia proximity in the white matter of 24-month-old mice. Scale bars, 20 μm. Bar plots show quantification of STAT1^+^ and STAT1^−^IBA1^+^ in proximity to CD8^+^ T cells and vice versa (3 sections per mouse were selected, and a total of 117 CD8^+^ T cells and 203 STAT1^+^IBA1^+^ microglia from 4 mice were analyzed). **b**, Quantification of the percentage of STAT1^+^IBA1^+^ microglia found in proximity to random cells compared with CD8^+^ T cells (*n* = 4 mice per group, ^**^P = 0.0052; data are mean ± s.e.m.; *P* value represents a two-sided paired Student’s *t*-test). **c**, Immunofluorescence staining and quantification of STAT1^+^IBA1^+^ microglia in the white matter of mice treated with anti-PD-1 and CTLA-4 (ICB) and isotype control antibodies (CTR) for 6 weeks starting at the age of 18 months (*n* = 3 mice per group, ^*^P = 0.0125; data are mean ± s.e.m.; *P* value represents a two-sided Student’s *t*-test). Scale bar, 20 µm. **d**, Immunofluorescence staining and quantification of STAT1^+^IBA1^+^ microglia in the white matter of 24-month-old wild-type and 24-month-old *CD8*^−*/*−^ mice (*n* = 4 mice per group, ^*^P = 0.0223; data are mean ± s.e.m.; *P* value represents a two-sided Student’s *t*-test). Source data

To validate the presence of IFN-responsive microglia in the aged white matter, we costained IBA1^+^ microglia by using antibodies against STAT1 and, in agreement with our scRNA-seq data, we detected STAT1^+^IBA1^+^ microglia in the white matter of 24-month-old mice (Fig. 4f). Notably, these cells were almost absent from the white matter of 3-month-old mice. Next, we studied the spatial relationship between STAT1^+^IBA1^+^ microglia and CD8^+^ T cells in aging white matter and found that they were frequently in close proximity (<20 µm) (Fig. 5a). Our analysis showed that STAT1^+^IBA1^+^ microglia are significantly more often found in close proximity to CD8^+^ T cells than randomly chosen DAPI^+^ cells (Fig. 5b). To determine whether the formation of STAT1^+^IBA1^+^ microglia depends on the function of adaptive immune cells, we first analyzed the effect of the treatment of 18-month-old mice with antibodies against the coinhibitory receptors CTLA-4 and PD-1. The number of STAT1^+^IBA1^+^ microglia increased within the corpus callosum by checkpoint blockage therapy (Fig. 5c). Next, we analyzed the number of STAT1^+^IBA1^+^ microglia in the white matter of 24-month-old *Rag1*^−/−^ mice, which revealed a reduction of STAT1^+^IBA1^+^ microglia number compared with control wild-type mice (Fig. 4f). Finally, we stained for STAT1^+^IBA1^+^ microglia in aged CD8^−/−^ mice. Again, 24-month-old mice deficient in functional CD8^+^ T cells had markedly lower numbers of STAT1^+^IBA1^+^ microglia compared with control wild-type mice (Fig. 5d). Together, these data show that CD8^+^ T cells not only induce an IFN-responsive oligodendrocyte but also microglia state in the aged white matter.

**Extended Data Fig. 8:**
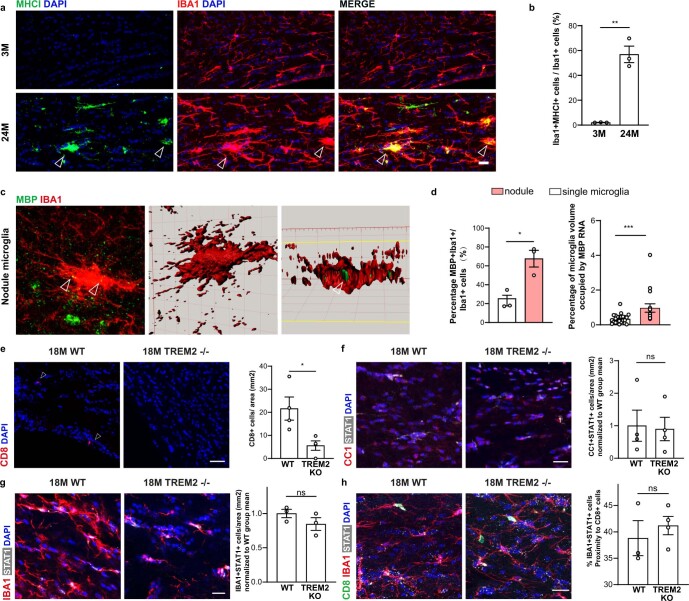
TREM2-independent formation of interferon-responsive oligodendrocytes and microglia in the aged white matter. **a**, Immunofluorescence staining and **b**, quantification of MHC1^+^IBA1^+^ microglia in the white matter of 3-month old and 24-month old mice. Arrow marks MHC1^+^ nodules (n = 3 mice per group, **P = 0.0012; data are means±s.e.m; *P* value represents a two-sided Student’s t-test). Arrows mark double positive microglia nodulces. **c**, Immunofluorescence combined with RNAscope of MBP mRNA (green) within IBA1^+^ microglia nodule (red). Clipped 3D images show MBP mRNA in a microglia nodule. Arrow mark MBP mRNA inside microglia. Scale bar, 20 µm, 3D rendering 10 µm. **d**, Quantification of percentage of IBA1^+^ single microglia and IBA1^+^ nodule with MBP mRNA inside (n = 3 mice per group, *P = 0.0160; data are means±s.e.m; *P* values represent a two-sided Student’s t-test). Quantification of percentage of IBA1^+^ volume occupied by MBP mRNA (single microglia, n = 26 cells, IBA1^+^ nodules, n = 30; ***P = 0.0004; data are means±s.e.m.; *P* value represents two-tailed Mann-Whitney test). **e**, Immunofluorescence staining and quantification of CD8^+^ T (red) cells in the white matter of 18-month old wild type and *Trem2*^−*/*−^ (n = 4 mice per group, *P = 0.0252; data are means±s.e.m; *P* value represents a two-sided Student’s t-test). Arrows indicate CD8^+^ T cells. Scale bar 20 µm. **f**, Immunofluorescence staining and quantification of STAT1^+^CC1^+^ oligodendrocytes in the white matter of 18-month old wild type and 18-month old *Trem2*^−*/*−^ mice (n = 4 mice per group; data are means±s.e.m; *P* value represents a two-sided Student’s t-test). Scale bar, 20 µm. **g**, Immunofluorescence staining and quantification of STAT1^+^IBA1^+^ microglia in the white matter of 18-month old wild type and 18-month old *Trem2*^−*/*−^ mice (n = 3 mice per group; data are means±s.e.m; *P* value represents a two-sided Student’s t-test). Scale bar, 20 µm. **h**, Immunofluorescence staining of CD8^+^ T cells and STAT1^+^IBA1^+^ microglia in the white matter of 18-month old wild type and 18-month old *Trem2*^−*/*−^ mice. Scale bars, 20 μm. Quantification of STAT1^+^IBA1^+^ microglia and CD8^+^ T cells proximity (<20 µm) in 18-month old wild type and *Trem2*^−*/*−^ mice (WT, n = 3; *Trem2*^−*/*−^, n = 4 mice; data are means ± s.e.m. *P* value represents a two-sided Student’s t-test). Source data

As our previous work identified the age-dependent formation of white matter-associated microglia, engaged in clearing myelin debris^18^, we asked whether these cells are required for lymphocyte-dependent IFN responses in aged white matter. This seemed plausible because white matter-associated microglia are defined by the activation of genes implicated in phagocytic activity, antigen processing and presentation, and they also express MHC-I (Extended Data Fig. 8a,b) and are enriched in *Mbp* transcripts (Extended Data Fig. 8c,d), reminiscent of microglia containing myelin transcripts previously detected in the brains of patients with multiple sclerosis^38^. As the triggering receptor expressed on myeloid cell 2 (TREM2) is required for the formation of white matter-associated microglia^18^, we analyzed *Trem2*^−/−^ mice to determine possible differences in the formation of IFN-responsive oligodendrocytes and microglia.

However, when aged *Trem2*^−/−^ mice were analyzed, we did not detect any significant differences in the number of STAT1^+^CC1^+^ oligodendrocytes or STAT1^+^IBA1^+^ microglia and also not in the proximity of STAT1^+^IBA1^+^ microglia to CD8^+^ T cells, even if there was a lower number of CD8^+^ T cells (Extended Data Fig. 8e–h), pointing to TREM2-independent mechanisms in the formation of IFN-responsive oligodendrocytes and microglia in aged white matter. However, aged *Trem2*^−/−^ mice suffer from slightly degenerating white matter and seizures^18^, which can cause innate immune reactions^39^, making the interpretation of the data more difficult.

### IFN-γ injection induces oligodendrocyte loss

**Fig. 6:**
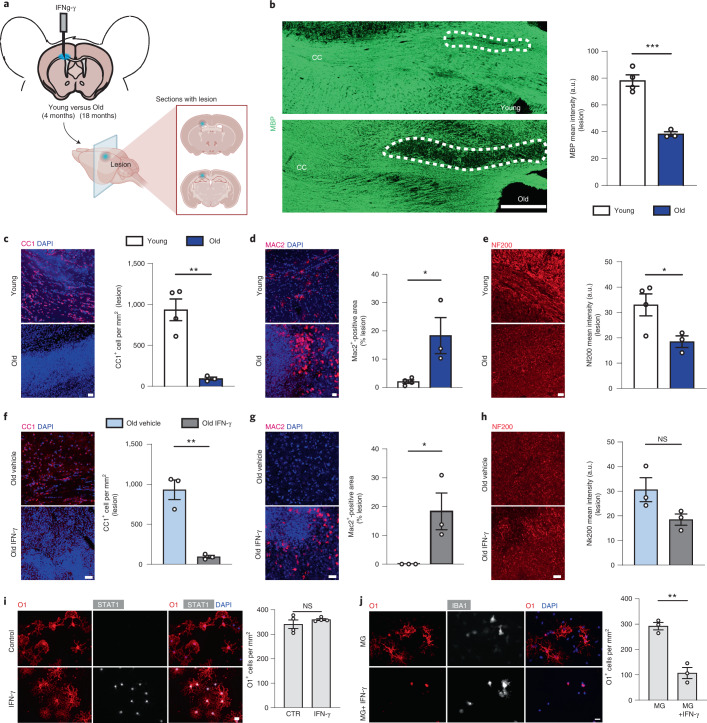
IFN-γ injections induce myelin and oligodendrocyte loss in aged mice. **a**, Diagram showing the model of IFN-γ injection in the corpus callosum (CC). The lesion area was identified by the positivity for monastral blue. **b**, Representative pictures of the CC after injection of 1 µl of a solution of 10 ng µl^−1^ of IFN-γ, 48 h postinjection. The intensity of the staining for MBP (green) was used to quantify the extent of demyelination (young, *n* = 4; old, *n* = 3; ^***^P = 0.0006; data are mean ± s.e.m.; *P* value represents a two-sided Student’s *t*-test). a.u., arbitrary units. **c**–**e**, Representative confocal images and quantifications of IFN-γ-mediated lesions in young and old mice, showing CC1^+^ oligodendrocytes (**c**), MAC2^+^ microglia (**d**) and NF200^+^ axons (**e**). The number of oligodendrocytes was expressed as number of cells per mm^2^; the amount of phagocytes was expressed as percentage area of the lesion occupied by MAC2^+^ cells; the extent of axonal damage was expressed as staining intensity of NF200 (young, *n* = 4; old, *n* = 3; CC1^+^, ^**^P = 0.0031; MAC2^+^, ^*^P = 0.0291; NF200^+^, ^*^P = 0.0451; data are mean ± s.e.m.; *P* values represent a two-sided Student’s t-test). Scale bar, 20 µm. **f**–**h**, Representative confocal images and quantifications of IFN-γ-mediated lesions in old mice and of the vehicle control showing CC1^+^ oligodendrocytes (**f**), MAC2^+^ cells (**g**) and NF200^+^ axons (**h**) (*n* = 3 mice per group, CC1^+^, ^**^P = 0.0025; MAC2^+^, ^*^P = 0.0451; data are mean ± s.e.m.; *P* values represent a two-sided Student’s *t*-test). **i**, Representative images of cultured oligodendrocytes (labeled for O1 in red and STAT1 in white) after treatment with IFN-γ for 24 h compared with control. Quantification of the number of oligodendrocytes after IFN-γ or vehicle treatment, expressed as number of O1^+^ cells per mm^2^ (*n* = 4 biological replicates per group, ^**^P = 0.0022; data are mean ± s.e.m.; *P* values represent a two-sided Student’s *t*-test). **j**, Representative images of oligodendrocytes cultured with microglia alone (MG) or microglia together with IFN-γ (MG+IFN-γ) and stained for O1 (oligodendrocytes) and IBA1 (microglia). Quantification of the number of oligodendrocytes, expressed as number of O1^+^ cells per mm^2^ (*n* = 4 biological replicates per group, ^**^P = 0.0022; data are mean ± s.e.m.; *P* values represent a two-sided Student’s *t*-test). Source data

**Extended Data Fig. 9:**
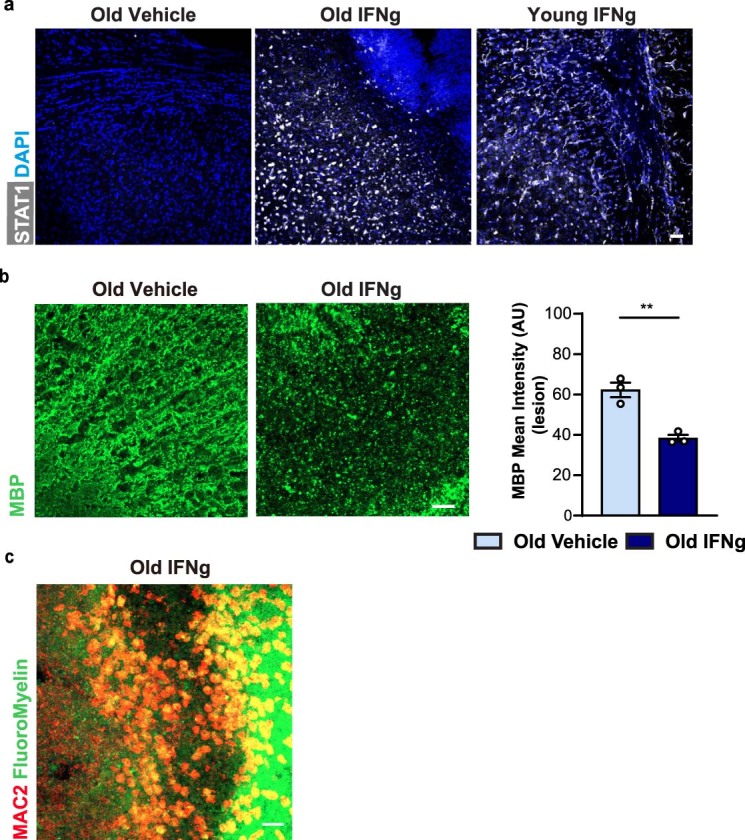
Characterization of IFN-γ-mediated lesions in the white matter of young and old mice. **a**, Representative confocal images of IFN-γ-mediated lesions in young and old mice and of the vehicle control stained for STAT1 (white) and DAPI (nuclei, blue). **b**, The data shown in Fig. 5 for IFN-γ-mediated lesions in 18-months old mice is shown here in comparison to vehicle control injections in 18-months old mice. Representative confocal images and quantifications of IFN-γ-mediated lesions in old mice and of the vehicle control showing MBP. The intensity of the staining for MBP was used to quantify the extent of demyelination (n = 3 mice per group, **P = 0.0040; data are means±s.e.m.; *P* value represents a two-sided Student’s t-test). **c**, Representative confocal images of IFN-γ-mediated lesions in old mice showing MAC2^+^ cells (red) with myelin (green) stained by FluoroMyelin. All scale bars, 20 μm. The experiments were repeated three times independently with similar results. Source data

Next, we performed experiments to determine the functional consequences of IFN on oligodendrocytes. Previous work has shown that CD8^+^ cytotoxic T lymphocytes in the aged brain produce IFN-γ^35^. To assess the impact of IFN-γ on oligodendrocytes, we stereotactically injected 10 ng of IFN-γ into the white matter of 4- and 18-month-old mice, which was sufficient to induce STAT1^+^CC1^+^ oligodendrocytes (Extended Data Fig. 9a). Strikingly, when lesions were analyzed 48 h postinjection in 4- and 18-month-old mice, we found that the aged mice contained more MAC2^+^ phagocytes loaded with myelin debris, displayed stronger reduction of CC1^+^ oligodendrocytes and showed more pronounced demyelination with loss of myelin basic protein (MBP) and a reduction of neurofilament labeling (Fig. 6a–e and Extended Data Fig. 9c). Control vehicle injections in 18-month-old mice did not induce such lesions (Fig. 6f–h and Extended Data Fig. 9b). Previous studies have shown that IFN-γ can induce oligodendrocyte cell death in vitro and in vivo^40–42^. To directly determine the response of IFN-γ on oligodendrocytes, we treated primary cultures of mouse oligodendrocytes with IFN-γ. We used IFN-γ in concentrations that triggered STAT1 expression, to determine whether the induction was sufficient to induce cell death. Immunofluorescence analysis indicated that the STAT1^+^ oligodendrocyte state did not affect cell viability in culture at this concentration (Fig. 6i). As IFN-γ is an important activator of microglia, we asked whether the cytotoxic effects of IFN-γ toward oligodendrocytes is mediated by microglia. Indeed, when oligodendrocytes were cocultured with microglia, a marked reduction of oligodendrocyte cell number was observed in the presence. but not in the absence, of IFN-γ (Fig. 6j). Thus, microglia can induce IFN-γ-mediated oligodendrocyte injury in vitro; however, other cells and mechanisms, such as direct CD8^+^ T-lymphocyte-mediated cytotoxicity, might contribute to oligodendrocyte reactions during aging in vivo.

## Discussion

White matter aging causes myelin degeneration, but how oligodendrocytes respond to aging is poorly defined. In the present study, we found that aging was associated with distinct oligodendrocyte responses, shown by the generation of a subpopulations of STAT1^+^/B2M^+^ and Serpina3n^+^/C4b^+^ oligodendrocytes and a reduction of oligodendrocyte density in aged white matter. We provided evidence that adaptive immune cells contributed to the cellular alterations that were associated with white matter aging. In both *Rag1*^−/−^ and *CD8*^−/−^ aged mice, the number of STAT1^+^ oligodendrocytes decreased and the total density of oligodendrocytes increased to a similar extent, providing evidence that functional CD8^+^ T cells are an important modifier of white matter aging. In addition, we identified a subpopulation of lymphocyte-dependent IFN-responsive microglia in aging white matter. These results show that adaptive immunity drives IFN-responsive cell states in aging white matter, but the exact link of CD8^+^ T cells, microglia and oligodendrocytes remains to be established. Previous work has shown that CD8^+^ T cells invading the aging brain exhibit high levels of IFN-γ, which drive IFN signaling within the neurogenic niche of the subventricular zone^35^. CD8^+^ T-cell-derived IFN-γ may also induce IFN responses in oligodendrocytes and microglia, but the contribution of other IFNs cannot be excluded^43^. One possible scenario is the secretion of IFN-γ by CD8^+^ T cells, which, in turn, polarizes microglia into an injurious phenotype. It is interesting to compare our results with a previous study using a viral model of encephalitis in mice, in which CD8^+^ T-cell-derived IFN-γ triggers acute loss of axosomatic connections^44^. In this model, phagocytes activated by neurons, which have been stimulated by CD8^+^ T-cell-derived IFN-γ triggers synapse loss^44^. Accordingly, it is conceivable that IFN-responsive oligodendrocytes actively recruit proinflammatory microglia as effector cells in aged white matter. Alternatively, CD8^+^ T-lymphocyte cytotoxicity occurs in a cognate T-cell receptor- and granzyme B-dependent manner, as shown for axons in the optic nerve^34^. In this context, the age-related induction of Serpina3n^+^ oligodendrocytes is of interest, because Serpina3n is an inhibitor of granzyme B that can dampen axon and myelin damage in autoimmune conditions^45^. Several studies have identified distinct populations of clonally expanded CD8^+^ T cells expressing the checkpoint inhibitor PD-1 in aged mice and humans^35,46^. We found that treatment of mice with antibodies against the checkpoint receptors, CTLA-4 and PD-1, resulted in an increase in the number of CD8^+^ T cells and IFN-responsive oligodendrocytes and microglia. These data could possibly explain why immune checkpoint inhibition in cancer causes behavioral and cognitive changes in some patients^47^.

The temporal sequence and causality of pathological processes contributing to white matter aging need to be established. As the deep white matter areas lie at the ends of the arterial circulation, they are particularly susceptible to decreases in blood flow and oxygenation, possibly contributing to increased vulnerability of aged white matter to hypoperfusion and aging-induced leaky blood–brain barrier^48,49^. Progressive vascular damage, induced by injury to myelinated fibers, may promote the infiltration of CD8^+^ T cells, thereby triggering harmful immune reactivity toward microglia and oligodendrocytes. A key question that remains to be established is how CD8^+^ T cells enter the brain and whether antigen recognition is necessary for this process. The clonal expansion of CD8^+^ T cells in aging provides evidence that they actively recognize antigen(s)^34,35^. It is unclear why CD8^+^ T cells migrate specifically to the central nervous system white matter. It is possible that they are attracted by myelin self-antigens, reminiscent of the T cells found in multiple sclerosis. The long-lived myelin proteins and lipids may accumulate oxidation and posttranslational modifications during aging that lead to their recognition as neoantigens when presented to T cells. Proof of principle that oligodendrocyte pathology can trigger adaptive autoimmune responses against myelin has been provided in a model of oligodendrocyte ablation^50^. However, passive mechanisms such as age-related changes in the migration of CD8^+^ T across a leaky blood–brain barrier, dural sinuses or choroid plexus are also conceivable. In addition, it remains to be clarified whether CD8^+^ T cells produce IFN-γ in the aging white matter, as has been shown for the stem cell niches of aging mice^35^.

Previous work has shown that oligodendrocytes are particularly sensitive to IFN-γ, because it can trigger oligodendroglial cell death and demyelination^40–42^. Strikingly, we find that this effect is highly pronounced in the aged brain, in which IFN-γ potently induces loss of oligodendrocytes and demyelinating injury. Possibly, the aging brain is primed toward IFN-γ due to age-associated, chronic, low-grade inflammation, so-called ‘inflammaging’^51^. Notably, OPCs respond to IFN-γ by inducing the antigen presentation pathway to activate T cells, which in turn can kill the OPCs as their target cells^28,30^. Immune responses in the aging brain are not limited to oligodendrocyte-lineage cells and myelinated axons. Previous work has shown T-cell infiltration in the aged brain, where the T cells impair the function of neuronal stem cells within the neurogenic niche^35,52,53^. Intriguingly, proliferation of neural stem cells was inhibited by IFN-γ, which was secreted from CD8^+^ T cells^35^. This mechanism is likely to be of functional relevance for the oligodendroglial lineage, because aging is not only associated with myelin degeneration but also with insufficient myelin renewal, a result of reduced capacity of OPCs to proliferate and differentiate^3,4,10,54,55^. Indeed, we found that myelin degeneration was not associated with an increase in OPC numbers in the aging brain.

As aging is the biggest factor for the most prevalent neurodegenerative diseases, it will be interesting to understand how the cellular alterations that occur in the white matter intermix with the pathology of these disorders. White matter changes and myelin alterations have been detected in Alzheimer’s disease and its mouse models^18,56,57^, which may contribute to disease pathology, including TREM2-dependent DAM signaling^16,17^; however, TREM2-independent glial responses have also been described^27,58^. As our previous work identified TREM2- and age-dependent, white matter-associated microglial responses, we analyzed the TREM2 dependence of IFN-responsive microglia but found instead a role of CD8^+^ T cells.

Our data emphasize the contribution of CD8^+^ T cells in triggering IFN-responsive cell states in the aging white matter, but it is likely that additional mechanisms contribute. In particular, prolonged exposure of nucleic acids to pattern-recognition, immune-sensing receptors can lead to inappropriate type I IFN release^59^. It is interesting that Aicardi–Goutieres syndrome, a prototype of an inherited disease with abnormal nucleic acid sensing and IFN induction, is associated with white matter pathology^59^. Likewise, deletion of ubiquitin-specific protease 18, a protein that negatively regulates STAT1 signaling, causes fatal activation of white matter microglia and myelin pathology^60,61^. Due to the extensive crosstalk between type 1 and type 2 IFN signaling pathways, future studies need to address their specific contribution within the different cell types during white matter aging.

Although the exact communication across CD8^+^ T cells, microglia and oligodendrocytes and the link to IFN signaling remain to be established, these results support the hypothesis that cytotoxic CD8^+^ T cells contribute to age-associated white matter decay.

## Methods

### Animals

The mouse lines used in the present study are the following: wild-type C57BL/6J mice were from Janvier Labs; *Trem2*^−/−^ mice^62^ on the C57BL/6J background were kindly provided by C. Haass, Laboratory of Neurodegenerative Disease Research, German Center for Neurodegenerative Diseases (DZNE), Munich; *Rag1*^−/−^ (B6.129S7-Rag1tm1Mom/J)^22^ and *Cd8*^−/−^ mice (B6.129S2-Cd8atm1Mak/J)^23^ and wild-type controls were on the C57BL/6J background. Experiments were performed with young, adult and aged mice (aged 3, 12, 18 and 24 months) as indicated in the figures and legends. Only aged mice that were on inspection healthy were used for experiments. Animals were randomly assigned to the different groups. Treatment with antibodies against PD-1 and CTLA-4 and their respective isotype controls was performed in 18-month-old mice; mice were injected intraperitoneally with a mix of both antibodies of concentration 10 mg kg^−1^ (PD-1) and 20 mg kg^−1^ (CTLA-4) twice a week for 6 weeks in total. All animal experiments were reviewed and overseen by the institutional animal use and care committee of the DZNE in Munich and the University Hospital in Würzburg. All animals were free from the most common mouse viral pathogens, ectoparasites, endoparasites and mouse bacterial pathogens harbored in research animals. The battery of screened infective agents met the standard health profile established in the animal facility in the DZNE animal housing facility. The mice were kept in groups in Greenline IVC GM500 plastic cages and were housed in a temperature-controlled environment (21 ± 2 °C) on a 12 h light:dark cycle with standard chow and water freely available. Water was provided in a water bottle, which was changed weekly. Cages were changed every week.

### Mice perfusion and cell isolation for Smart-seq2

Four young (3-month-old) and four old (24-month-old) male C57BL/6J mice were deeply anesthetized and perfused with cold phosphate-buffered saline (PBS; Sigma-Aldrich, catalog no. D8537). Each brain was carefully removed and individually microdissected under a dissection microscope; gray matter was isolated from the frontal cortex and white matter was carefully isolated from the optic tract, medial lemniscus and corpus callosum (attached gray matter and choroid plexus were removed). We used our previously established isolation protocol^63^ using gentleMACS with the Neural Tissue Dissociation Kit (Papain; Miltenyi Biotec) and a final concentration of 45 mM actinomycin D (Act-D, Sigma-Aldrich, catalog no. A1410). Subsequently, cells were blocked with mouse FcR-blocking reagent (CD16/CD32 Monoclonal Antibody, eBioscience, catalog no. 14-0161-82,1100), stained with antibodies against CD11b (PE/Cy7, M1/70, eBioscience, catalog no. 48-0451-82, 1:200) and afterward washed with PBS. Before sorting, the cell suspensions were stained by the live/dead marker SYTOX Blue (final concentration 1 µM). Viable (SYTOX Blue-negative) nonmyeloid single cells (CD11b^−^ cells) were sorted by flow cytometry (Sony, catalog no. SH800). Single cells were sorted into 96-well plates filled with 4 ml of lysis buffer containing 0.05% Triton X-100 (Sigma-Aldrich) and ERCC (External RNA Controls Consortium) RNA spike-in Mix (Ambion, Life Technologies; 1:24,000,000 dilution), 2.5 mM oligo(dT), 2.5 mM dNTP and 2 U ml^−1^ of recombinant RNase inhibitor (Clontech), then spun down and frozen at −80 °C. Plates were thawed and libraries prepared as described below.

### Library preparation for Smart-seq2

The 96-well plates containing the sorted single cells were first thawed and then incubated for 3 min at 72 °C and thereafter immediately placed on ice. To perform reverse transcription (RT) we added to each well a mix of 0.59 μl of H_2_O, 0.5 μl of SMARTScribe Reverse Transcriptase (Clontech), 2 μl of 5× First Strand buffer, 0.25 μl of Recombinant RNase Inhibitor (Clontech), 2 μl of Betaine (5 M Sigma), 0.5 μl of dithiothreitol (100 mM), 0.06 μl of MgCl_2_ (1 M, Sigma-Aldrich) and 0.1 μl of template-switching oligos (TSOs) (100 μM, AAGCAGTGGTATCAACGCAGAGTACrGrG + G). Next, RT reaction mixes were incubated at 42 °C for 90 min, followed by 70 °C for 5 min and 10 cycles of 50 °C for 2 min and 42 °C for 2 min, finally ending with 70 °C for 5 min for enzyme inactivation. Preamplification of complementary DNA was performed by adding 12.5 μl of KAPA HiFi Hotstart 2× (KAPA Biosystems), 2.138 μl of H_2_O, 0.25 μl of ISPCR primers (10 μM, 5′-AAGCAGTGGTATCAACGCAGAGT-3′) and 0.1125 μl of lambda exonuclease under the following conditions: 37 °C for 30 min, 95 °C for 3 min, 23 cycles of 98 °C for 20 s, 67 °C for 15 s and 72 °C for 4 min, and a final extension at 72 °C for 5 min. Libraries were then cleaned using AMPure bead (Beckman-Coulter) clean-up at a 0.7:1 beads:PCR product ratio. Libraries were assessed using Bio-analyzer (Agilent, catalog no. 2100), using the High Sensitivity DNA analysis kit, and also fluorometrically using Qubit’s DNA HS assay kits and a Qubit 4.0 Fluorometer (Invitrogen, Life Technologies) to measure the DNA concentrations. Further selection of samples was performed via quantitative PCR assay against ubiquitin transcript Ubb77 (primer 1 5′-GGAGAGTCCATCGTGGTTATTT-3′; primer 2 5′-ACCTCTAGGGTGATGGTCTT-3′; probe 5′-/5Cy5/TGCAGATCTTCGTGAAGACCTGAC/3IAbRQSp/−3′) measured on a LightCycler 480 Instrument II (Roche). Samples were normalized to 160 pg µl^−1^. Sequencing libraries were constructed using in-house-produced Tn5 transposase33. Libraries were barcoded and pooled then underwent three rounds of AMPure bead (Beckman-Coulter) clean-up at a 0.8:1 ratio beads:library. Libraries were sequenced 2× 150 reads bp paired-end on Illumina HiSeq4000 to a depth of 3 × 105–4 × 105 reads per sample.

### Processing, quality control and analyses of Smart-seq2 scRNA-seq data

BCL files were demultiplexed with the bcl2fastq software from Illumina. After quality control with FastQC, reads were aligned using rnaSTAR^64^ to the GRCm38 (mm10) genome with ERCC synthetic RNA added. Read counts were collected using the parameter ‘quantMode GeneCounts’ of rnaSTAR and the unstranded values. Quantitative criteria were used to filter out low-quality cells as shown in Extended Data Fig. 1b. We observed the distribution of all samples for each quality metrics and defined thresholds to remove outliers or samples with abnormal values. In the same order as in Extended Data Fig. 1b, we considered the number of reads per sample (≥20,000 and ≤4 × 10^6^), the number of genes per sample (≥1,000 and ≤6,500), the average sequence read length after trimming (≥180 and ≤200), the mismatch rate during alignment (≥0.15 and ≤0.5), the percentage of uniquely mapped reads (≥68 and ≤100), the percentage of multimapped reads (≥2.3 and ≤7.7), the percentage of reads considered too short (≥0 and ≤17), the percentage of ERCCs (≥0 and ≤0.011) and the percentage of mitochondrial genes (≥0 and ≤0.006). From 2,650 single cells, 2,538 passed quality control. From that point, Seurat v.3.2.3R package was utilized^65^. Gene expressions were normalized using the SCTransform function (3,000 variable features) within Seurat. The first eight PCs were selected based on the elbow plot and heatmap of PC embeddings and used for downstream analysis steps. Cell-type clusters were identified using the Louvain algorithm and annotated by canonical cell-type markers (Extended Data Fig. 1c,d). Oligodendrocytes (2,413 single cells) were extracted and analyzed separately. After processing with SCTransform (2,000 variable features), the first 10 PCs were considered for downstream analyses. Unbiased clustering was performed using the Louvain algorithm that led to the identification of the four aforementioned oligodendrocyte populations. Gene sets of clusters 1, 2, 3 and 4 were defined by using the FindMarkers function with a threshold of avg_log_2_(fold-change) (avg_log_2_(FC)) > 1 (Fig. 1g). GO analyses were performed with the DAVID annotation tool^66^, STRING^67^ and Metascape^68^.

### Mice perfusion and cell isolation for 10× genomic experiments

For 10× genomic experiments, mice were deeply anesthetized and perfused with cold PBS. Each brain was removed, individually microdissected under a dissection microscope and dissociated in the same way as described above (10× mice information is provided in Supplementary Table 2). To collect enough cells for loading on to the 10× Genomics Chromium chip, two gray matter/white matter tissue samples were combined into one sample. After tissue dissociation, SYTOX Blue-negative cells were sorted into a 2-ml Eppendorf tube with 1 ml of RPMI + 5% fetal bovine serum (FBS). Sorted cells were centrifuged at 300*g* for 10 min at 4 °C. Cell pellets were resuspended in 0.04% bovine serum albumin (BSA) + PBS catching medium at a concentration of 700–900 cells per μl.

### Library preparation for 10× genomic experiments

Single-cell suspensions were loaded on to the Chromium Single Cell Controller using the Chromium Single Cell 3′ Library & Gel Bead Kit v.3.1 (10× Genomics) chemistry following the manufacturer’s instructions. Sample processing and library preparation were performed according to the manufacturer’s instructions using AMPure beads (Beckman-Coulter). Libraries were sequenced on the DNBSEQ Sequencing System (BGI group).

### Preprocessing and analyses of 10× data

Fastq files were processed with Cell Ranger v.3.0.2 (wild-type aging), 4.0.0 (Rag1KO 1st batch) and 6.1.2 (Rag1KO 2nd batch). From that point, the Seurat v.3.2.3R package^65^ was used for downstream analyses. Unless stated otherwise, all gene expression matrices were filtered with the parameters ‘min.cells=3’, ‘min.genes=200’ and ‘mitochondrial percentage>0.10’ (Extended Data Fig. 6c), removing cells with <200 genes and mitochondrial gene percentage >10% and keeping genes with expression in at least 3 cells. Further sets of filters are explained in detail for each sample.

#### Wild-type aging datasets

The two batches of libraries (Supplementary Table 2) were processed separately. For the first batch, expression matrices were filtered by number of unique molecular modifiers (UMIs) (<30,000) and genes (<6,000). Processed data were normalized with the SCTransform function (variable.features.rv.th=1.4) and the top 9 PCs were selected for downstream analyses on inspection. Major cell types were identified using Louvain clustering and canonical cell-marker expression. Oligodendrocytes were extracted to be analyzed separately. The oligodendrocyte subset dataset was processed with SCTransform (variable.features.rv.th=1.5), principal component analysis (ten PCs) and Louvain clustering as explained earlier. In addition, *Gm42418* and *AY036118* genes were removed from the expression matrix because they indicate ribosomal RNA contamination^69^.

The samples in the second batch were analyzed similarly; by extracting the oligodendrocytes after processing the libraries by quality control (QC) filtering, normalization, dimensionality reduction, clustering and cell-type annotation. QC filtering was done by number of UMIs (<50,000) and genes (<8,000). The filtered expression matrix was normalized by SCTransform (variable.features.rv.th=1.4 and regression by ‘mitochondrial percentage’). The top 30 PCs were picked for downstream analyses. The oligodendrocyte subset dataset was again put through the same processing steps: SCTransform (top 750 variable genes) normalization, preceding downstream analyses conducted with the top 10 PCs.

To avoid batch-specific effects while still preserving biological variability, the sequenced libraries (four sequencing runs from eight animals) were integrated using the Seurat 3 CCA integration workflow^70^. Both the dataset with all cell types and the oligodendrocyte subset dataset were integrated using the integration steps tailored for SCTransform-normalized datasets. For the dataset with all cell types, the first 30 PCs were selected for the downstream analyses. Major cell types were identified using Louvain clustering and canonical cell-marker expression (Extended Data Fig. 1e,f).

For the oligodendrocyte dataset integration, the top 750 most variable genes and the top 20 PCs were selected for the ‘anchoring’. After the integration, the top 10 PCs were used for downstream steps and unbiased clustering with a resolution of 0.5 identified the aforementioned 7 oligodendrocyte populations.

#### Rag1KO datasets

Samples from the two batches were analyzed separately, similar to the steps described for the wild-type aging datasets. For the first batch, expression matrices were filtered by number of UMIs (<20,000). Processed data were normalized using the SCTransform function (variable.features.rv.th=1.4 and regression by ‘mitochondrial percentage’). The top 30 PCs were selected for downstream analyses and major cell types were identified using Louvain clustering and canonical cell-marker expression. A small cluster with high microglial-marker gene expression was removed. Oligodendrocytes were extracted and analyzed separately before integration. *Gm42418* and *AY036118* genes were removed from the expression matrix to prevent technical artifacts. In addition, a small cluster with high microglial-marker gene expression were removed. The same normalization parameters were applied: variable.features.rv.th=1.4 and regression by ‘mitochondrial percentage’. The second batch of samples (both all cell types and oligodendrocyte subsets) were analyzed in the same way with the exception of filtering parameters (number of UMIs <25,000 and genes <8,000).

#### Integration of wild-type and Rag1KO datasets

The Seurat v.3 RPCA integration method was used to analyze wild-type and Rag1KO samples together. Previously described four batches of oligodendrocytes subsets (eight sequencing runs in total, four per genotype) were integrated (Fig. 3d and Extended Data Fig. 6c). The 500 most variable genes from each batch and the top 20 PCs were used in the ‘anchoring’ (k.anchor=3) of the Seurat objects. Postintegration, the top 15 PCs were selected for downstream analyses. Unbiased clustering identified the same oligodendrocyte populations as previously described.

To analyze wild-type and Rag1KO microglia, our previously described microglia dataset^18^ was integrated together with the microglia from our Rag1KO libraries. Four batches of microglia subsets (eight sequencing runs in total, four per genotype) were integrated (Fig. 4a and Extended Data Fig. 6c). The 750 most variable genes from each batch and the top 15 PCs were used in the ‘anchoring’ (k.anchor=4, max.features=100) of the Seurat objects. Postintegration, the top 15 PCs were selected for downstream analyses. Unbiased clustering identified the same microglial populations in Safaiyan et al.^18^ with addition to IFN-responsive microglia.

Datasets generated in the present study and external datasets (12 sequencing runs, 4 from Safaiyan et al.^18^, 8 from the present study) were integrated using RPCA (Fig. 3a). The 3,000 most variable genes and default parameters for other functions were used in the ‘anchoring’. The first 30 PCs were selected for clustering analysis and major cell types were identified using canonical marker genes.

#### Integration of CD8 T-cell datasets

Processed gene expression matrices and metadata of brain (FACS), kidney (droplet), lung (droplet) and spleen (droplet) datasets from the mouse aging single-cell transcriptomic atlas^36^ were downloaded. After filtering based on gene expression and age, only CD8^+^ T cells from 21- and 24-month-old samples were kept. The processed gene expression matrix and metadata were provided on request by the authors for the Groh et al. dataset^34^. These five datasets were integrated with the RPCA workflow for log(normalized datasets); the 500 most variable genes and the top 10 PCs were used in the anchoring steps. The integrated dataset was scaled with default parameters and the top ten PCs were used to calculate the UMAP.

All differential gene expression analyses were conducted using the FindMarkers function with Wilcoxon’s rank-sum test. The scCODA v.0.1.6 package was used for compositional analysis of the single-cell data. The false discovery rate (FDR) value was set to 0.4 to be able to detect subtle yet biologically relevant changes, as described by the authors in their documentation. In all boxplots, the central line denotes the median, boxes represent the interquartile range (IQR) and whiskers show the distribution except for outliers. Outliers are all points outside 1.5× the IQR.

### Immunohistochemistry

Animals were anesthetized by 10 mg ml^−1^ of ketamine and 1 mg ml^−1^ of xylazine solution intraperitoneally and perfused transcardially with 4% paraformaldehyde (PFA). Postfixation of brain tissue was done in 4% PFA overnight. Then the brain tissue was further cryoprotected in 30% sucrose in PBS for 24 h. After freezing the tissue on dry ice using Tissue-Tek O.C.T, 30-μm coronal sections were cut using a cryostat Leica CM 1900. Free-floating sections were collected in a solution containing 25% glycerol and 25% ethylene glycol in PBS. The sections were rinsed with 1× PBS containing 0.2% Tween-20 and permeabilized in 0.5% Triton X-100 for 30 min. Fab fragment goat anti-mouse immunoglobulin G (1:100, Dianova) was added for 1 h at room temperature to block endogenous mouse tissue immunoglobulins. After a brief wash the sections were blocked for 1 h at room temperature in a solution containing 2.5% fetal calf serum, 2.5% BSA and 2.5% fish gelatin in PBS. Primary antibodies, diluted in 10% blocking solution, were incubated overnight at 4 °C. On the following day, sections were incubated with secondary antibodies, diluted in 10% blocking solution, for 2 h at room temperature. The sections were washed with PBS followed by DAPI incubation in 1× PBS for 10 min and mounted. The following antibodies were used: mouse anti-APC (Millipore, catalog no. OP80-100UG, 1:100), rabbit anti-B2m (abcam, catalog no. ab75853-100ul, 1:100), rabbit anti-STAT1 (Cell Signaling Technology, catalog no. 14994S, 1:250), rat anti-CD8 (Promega, catalog no. 100702, 1:100), rabbit anti-Iba1 (Wako, catalog no. 234 004, 1:250), goat anti-Serpina3n (Bio-Techne, catalog no. AF4709, 1:100), rat anti-C4b (Thermo Fisher Scientific, catalog no. MA1-40047,1:25), rabbit anti-Olig2 (Millipore, catalog no. AB9610, 1:250), mouse anti-Gstp (BD, catalog no. 610719, 1:250), rat anti-Mac2 (BioLegend, catalog no. 125402, 1:250), chicken anti-MBP (Thermo Fisher Scientific, catalog no. PA1-10008, 1:1,000), chicken anti-neurofilament heavy polypeptide (abcam, catalog no. ab4680, 1:400), anti-PDGF-Ralpha (R&D Systems, catalog no. 1:100), goat anti-CD69 (R&D Systems,1:100), goat anti-PD-1 (R&D Systems,1:100), rabbit anti-LAG-3 antibody (Abcam, 1:100), AF1062FM green fluorescent myelin stain (Thermo Fisher Scientific, catalog no. F34651, 1:400), anti-mouse 555 (Thermo Fisher Scientific, catalog no. A-21422, 1:500), anti-mouse 647 (Thermo Fisher Scientific, catalog no. A-21235, 1:500), anti-mouse 488 (Thermo Fisher Scientific, catalog no. A-21202, 1:500), anti-rabbit 555 (Thermo Fisher Scientific, catalog no. A-21428, 1:500), anti-rabbit 488 (Thermo Fisher Scientific, catalog no. A-11008,1:500), anti-rat 555 (Thermo Fisher Scientific, catalog no. A-21434, 1:5600), anti-goat 555 (Thermo Fisher Scientific, catalog no. A-32116, 1:500), donkey anti-rat 488 (Thermo Fisher Scientific, 1:500), donkey anti-goat 555 (Thermo Fisher Scientific, 1:500) and donkey anti-rabbit 647 (Thermo Fisher Scientific, 1:500). For CC1, B2m, Gstp, OLIG2, STAT1 and Serpina3n staining, antigen retrieval protocol using citrate buffer (10 mM, pH 6) was performed on free-floating sections, followed by a staining protocol as mentioned above. For CD8, STAT1 and CC1/IBA1 combined staining, the sections were permeabilized with 0.5% Triton X-100 for 30 min at room temperature and blocked in 5% goat serum, and primary antibodies, diluted in 10% blocking solution, were added and incubated overnight at 4 °C with 0.5% Triton X-100 for 30 min at room temperature and blocked in 5% goat serum; secondary antibodies include goat anti-rabbit IgG antibody (H + L), biotinylated (Vector Laboratories, 1:200), goat anti-rat 488 and goat anti-mouse 647 (Invitrogen, 1:500) for 1 h at room temperature. Sections were then washed with PBS and incubated with streptavidin 555 (Invitrogen, 1:500). To determine the proximity of CD8^+^ T cells to STAT1^+^CC1^+^ oligodendrocytes (IROs), CD8^+^ T cells (or random DAPI^+^ cells) were selected manually, from which a circle with a 20-µm radius was drawn from the center of the cell via ImageJ automatically. We then quantified the percentage of STAT1^+^CC1^+^ oligodendrocytes located within that circle. Conversely, we proceeded similarly but took oligodendrocytes as a reference and quantified the T cells within a circle with a 20-µm radius. The same quantification method was applied to determine the proximity of CD8^+^ T cells to STAT1^+^IBA1^+^ microglia (IRM). Images were acquired via a Leica TCS SP5 confocal microscope or with an LSM900 Zeiss microscope and were processed and analyzed with ImageJ 1.41 image-processing software.

### Correlated light and electron microscopy

Mice were perfused by 4% PFA (electron microscope (EM) grade, Science Services) in PBS (pH 7.4), the brain dissected and vibratome sectioned coronally at 100-µm thickness. Every second section was subjected to immunohistochemistry, whereas the remaining sections were postfixed with 2.5% glutaraldehyde in 0.1 M cacodylate buffer (Science Services) for potential EM. The method of immunohistochemistry for Iba1 staining was described as above. Sections were assessed by fluorescence imaging for sites of Iba1 enrichment in the corpus callosum, indicative of microglial nodules. The adjacent EM section was selected and subjected to a standard rOTO contrasting procedure. After postfixation in 2% osmium tetroxide (Electron Microscope Services), 1.5% potassium ferricyanide (Sigma-Aldrich) in 0.1 M sodium cacodylate, staining was enhanced by reaction with 1% thiocarbohydrazide (Sigma-Aldrich) for 45 min at 40 °C. The tissue was washed in water and incubated in 2% aqueous osmium tetroxide, washed and further contrasted by overnight incubation in 1% aqueous uranyl acetate at 4 °C and 2 h at 50 °C. Samples were dehydrated in an ascending ethanol series and infiltration with LX112 (LADD). We serially sectioned the tissue at 200-nm thickness on to carbon nanotube (CNT) tape (Science Services) on an ATUMtome (Powertome, RMC) using a 35° ultra-diamond knife (Diatome). CNT tape stripes were assembled on to adhesive carbon tape (Science Services) attached to 4-inch silicon wafers (Siegert Wafer) and grounded by adhesive carbon tape strips (Science Services). EM micrographs were acquired on a Crossbeam Gemini 340 SEM (Zeiss) with a four-quadrant backscatter detector at 8 kV. Overview scans were taken at 200-nm lateral and higher resolution scans at 20-nm lateral resolution. Serial section data were aligned by a sequence of automatic and manual processing steps in Fiji TrakEM2 (ref. ^71^) and relevant regions selected.

### Subregional localization analysis

To compare the difference between frontal white matter and medial white matter, coronal sections with corpus callosum were divided into two groups following the *Allen Mouse Brain Atlas*: the white matter in the front of the brain, which does not contain lateral ventricles, was defined as ‘frontal white matter’ and white matter from sections that have lateral ventricles is defined as ‘medial white matter’. For each quantification, two brain sections were selected from each group of three to four mice. For statistical analysis the paired Student’s *t*-test was used.

### RNAscope in situ hybridization

RNAscope in situ hybridization assay was applied to detect *Mbp* mRNA in the brain cryosections prepared from aged wild-type mice as performed previously^18^. The assay was performed using a commercially available RNAscope Multiplex Fluorescent Detection Reagent v.2 (Advanced Cell Diagnostics) kit and the manufacturer’s instructions were followed. Briefly, 30-μm cryosections were fixed on superfrost plus slides; they were pretreated with hydrogen peroxide for 10 min at room temperature and then with antigen retrieval reagent (5 min of boiling) to unmask the target RNA. After applying Protease III on the sections for 30 min at 40 °C, probe hybridization was done by incubating sections in mouse *Mbp* probe assigned to channel 3, diluted 1:50 in probe diluent, for 2 h at 40 °C. Afterwards, signal amplification and detection were performed according to the kit’s instruction. Signal detection was done using Opal dyes (Opal520-green) diluted 1:3,000 in tyramide signal amplification (TSA) buffer. To visualize microglia, after in situ hybridization, an immunohistochemistry assay was performed using Iba1 antibody (Wako, 1:1,000). After washing with 1× PBS, sections were incubated for 30 s with 1× TrueBlack to remove autofluorescence background. The nuclei of cells were counterstained with DAPI and mounted. Images were acquired via a Leica TCS SP5 confocal microscope or with an LSM900 Zeiss microscope and were processed and analyzed with ImageJ 1.41 image-processing software.

### Cell culture

OPCs were prepared from P8 C57BL/6J mouse brains by immunopanning^72^. Briefly, brains were dissociated to single-cell suspension, which was passed through two negative-selection plates coated with BSL1 to remove microglia. The remaining cell suspension was then incubated in a positive-selection plate coated with anti-CD140a antibodies. The attached cells were collected by accutase and cultured on poly(l-lysine)-coated coverslips in proliferation medium containing Dulbecco’s modified Eagle’s medium (DMEM; Thermo Fisher Scientific, catalog no. 41965), Sato Supplement, B-27 Supplement, GlutaMAX, Trace Elements B, penicillin–streptomycin, sodium pyruvate, insulin, *N*-acetyl-l-cysteine, d-biotin, forskolin, ciliary neurotrophic factor (CNTF), platelet-derived growth factor (PDGF) and neurotrophin-3 (NT-3). Primary microglia cultures were prepared from p11 C57BL/6J mouse brains. The brains were homogenized to a single-cell suspension using the neural tissue dissociation kit (Miltenyi Biotech, catalog no. 130-092-628) and by filtering the homogenate through a 70-μm cell strainer to remove tissue debris. Then, the cells were incubated with magnetic beads against CD11b and the solution was passed through a magnetic column. Microglia were flushed and plated in DMEM supplemented with 10% bovine calf serum, 10 ng ml^−1^ of monocytic colony-stimulating factor, 1% penicillin–streptomycin and 1% glutamate for 4–7 d before using them for experiments. For the coculture experiments, OPCs were cultured in differentiation medium containing DMEM (Thermo Fisher Scientific), Sato Supplement, B-27 Supplement, GlutaMAX, Trace Elements B, penicillin–streptomycin, sodium pyruvate, insulin, *N*-acetyl-l-cysteine, d-biotin, forskolin, CNTF and NT-3. After 1.5 d, when OPCs had differentiated into oligodendrocytes, microglia were collected after scraping, counted and plated with oligodendrocytes. After 6 h, IFN-γ (Millipore, catalog no. IF005) in 5 mM phosphate buffer, pH 8.0 containing 0.1% BSA was diluted 1:5,000 in the coculture to a final concentration of 0.1 ng µl^−1^. For the control, phosphate buffer containing BSA was diluted in the same way. After 2 d, the coculture was fixed and analyzed by immunocytochemistry. Briefly, the cells were permeabilized with 0.1% Triton X-100 for 1 min and blocked in 10% blocking solution for 1 h. The cells were then incubated with primary antibodies overnight at 4 °C, washed twice in PBS and incubated for 1 h at room temperature with secondary antibodies. Oligodendrocytes were stained for O1 (mouse hybridoma, 1:5), microglia for IBA1 (Wako, catalog no. 234 004, 1:250) and nuclei were stained with DAPI. After mounting, the cells were imaged on a Leica DMI6000 widefield microscope (×20, 0.4 numerical aperture, air objective) and analyzed using Fiji.

### Stereotactic injection in the corpus callosum

A solution of 10 ng µl^−1^ of IFN-γ was prepared by mixing IFN-γ with sterile 1× PBS. Monastral blue (Sigma-Aldrich, catalog no. 274011; autoclaved and sterile filtered) was added to a final concentration of 0.03% just before injection to identify the lesion area during tissue processing. Mice were anesthetized with an intraperitoneal injection of MMF solution (0.5 mg medetomidin per kg (body weight), 5 mg midazolam per kg (body weight) and 0.05 mg fentanyl per kg (body weight)). Then the head was shaved and the eyes were protected with bepanthene cream (Bayer, catalog no. 1578847). A small incision was performed in the skin to expose the skull. The mouse was positioned into a stereotactic injection apparatus and a small hole was drilled at the injection coordinates: *X*, ±0.55 mm; *Y*, −1.22 mm (from the bregma). The glass capillary containing the IFN-γ solution or the control solution (PBS) was then lowered to *Z* of −1.25 mm from bregma, and 1 µl was injected at a rate of 100 nl min^−1^. Then, 3 min after the delivery of the solution, the capillary was slowly retracted. The mouse was then injected with 0.05 mg of buprenorphin per kg (body weight) and the skin wound was sutured. Anesthesia was terminated by the injection of the antagonist solution, containing 2.5 mg kg^−1^ of atipamezol, 1.2 mg kg^−1^ of naloxone and 0.5 mg kg^−1^ of flumazenil. After 48 h, the animals were perfused transcardially with 4% PFA. Postfixation of brain tissue was done in 4% PFA overnight. Then the brain tissue was further cryoprotected in 30% sucrose and PBS for 24 h. The brain lesions were sectioned and stained using the same method as used in Immunohistochemistry.

### Statistical analysis

For immunohistochemistry analysis three to six sections from each animal were analyzed. Data are shown as mean ± s.e.m. No statistical methods were used to predetermine sample sizes, but our sample sizes are similar to those generally employed in the field^16–20^. Each dot represents one animal. Normal distribution of the samples was tested using the Shapiro–Wilk test. For statistical analysis, paired or unpaired Student’s *t*-test, or the Mann–Whitney *U*-test, was used to compare two groups. Two-sided, one-way analysis of variance (ANOVA) followed by post hoc Tukey’s test was used for multiple comparisons. Test were chosen according to their distribution. In all tests a *P* value <0.05 was considered significant with ^*^P < 0.05, ^**^P < 0.01 and ^***^P < 0.001. Statistical analyses were done using GraphPad Prism (GraphPad Software, Inc.). Data acquisition and analysis were performed in a blinded manner. No animals were excluded from the analyses.

### Reporting summary

Further information on research design is available in the Nature Research Reporting Summary linked to this article.

## Online content

Any methods, additional references, Nature Research reporting summaries, source data, extended data, supplementary information, acknowledgements, peer review information; details of author contributions and competing interests; and statements of data and code availability are available at 10.1038/s41593-022-01183-6.

## Supplementary information

Reporting Summary

Supplementary Table 1 A complete list of mice used for Smart-seq2 experiments.

Supplementary Table 2 A complete list of mice used for 10x experiments.

Supplementary Table 3 GO enrichment details for the cluster marker gene sets.

## Data Availability

The datasets we used (scRNA-seq) are deposited at the Gene Expression Omnibus (National Center for Biotechnology Information) under accession no. GSE202579. External datasets used in the present study include data from accession nos. GSE166548, GSE138891 and GSE132042. Source data are provided with this paper.

## Source data

Source Data Fig. 2 Statistical source data.
Source Data Fig. 3 Statistical source data.
Source Data Fig. 4 Statistical source data.
Source Data Fig. 5 Statistical source data.
Source Data Fig. 6 Statistical source data.
Source Data Extended Data Fig. 2 Statistical source data.
Source Data Extended Data Fig. 3 Statistical source data.
Source Data Extended Data Fig. 5 Statistical source data.
Source Data Extended Data Fig. 7 Statistical source data.
Source Data Extended Data Fig. 8 Statistical source data.
Source Data Extended Data Fig. 9 Statistical source data.

## Code availability

The R code used for the analyses can be found at the github web-page: https://github.com/ISD-SystemsNeuroscience/Aging_Oligos_Microglia.
