# Supplementary material for: CD8+ T cells induce interferon-responsive oligodendrocytes and microglia in white matter aging
Source: Nat Neurosci. 2022 Oct 24;25(11):1446–57. doi: 10.1038/s41593-022-01183-6 (PMC9630119; doi:10.1038/s41593-022-01183-6)
Supplement: Supplementary file 1 — Reporting Summary [file 41593_2022_1183_MOESM1_ESM.pdf]

nature portfolio

Corresponding author(s): Mikael Simons, Ozgun Gokce

Last updated by author(s): Sep 9, 2022

Reporting Summary

Nature Portfolio wishes to improve the reproducibility of the work that we publish. This form provides structure for consistency and transparency in reporting. For further information on Nature Portfolio policies, see our Editorial Policies and the Editorial Policy Checklist.

Statistics

For all statistical analyses, confirm that the following items are present in the figure legend, table legend, main text, or Methods section.

n/a	Confirmed	
☐	☒	The exact sample size (*n*) for each experimental group/condition, given as a discrete number and unit of measurement
☐	☒	A statement on whether measurements were taken from distinct samples or whether the same sample was measured repeatedly
☐	☒	The statistical test(s) used AND whether they are one- or two-sided<br>*Only common tests should be described solely by name; describe more complex techniques in the Methods section.*
☒	☐	A description of all covariates tested
☐	☒	A description of any assumptions or corrections, such as tests of normality and adjustment for multiple comparisons
☐	☒	A full description of the statistical parameters including central tendency (e.g. means) or other basic estimates (e.g. regression coefficient) AND variation (e.g. standard deviation) or associated estimates of uncertainty (e.g. confidence intervals)
☐	☒	For null hypothesis testing, the test statistic (e.g. *F*, *t*, *r*) with confidence intervals, effect sizes, degrees of freedom and *P* value noted<br>*Give P values as exact values whenever suitable.*
☒	☐	For Bayesian analysis, information on the choice of priors and Markov chain Monte Carlo settings
☒	☐	For hierarchical and complex designs, identification of the appropriate level for tests and full reporting of outcomes
☐	☒	Estimates of effect sizes (e.g. Cohen's *d*, Pearson's *r*), indicating how they were calculated

Our web collection on statistics for biologists contains articles on many of the points above.

Software and code

Policy information about availability of computer code

Data collection

1. Confocal images: all images of mouse brain sections were acquired using a Leica TCS SP5 or Zeiss LSM900 confocal microscope and were processed with Imaris (64x version 9.2.0) and ImageJ 1.41 image processing softwares. A Leica DMI6000 widefield microscope was used for cell culture experiments

2. Single-cell RNA-seq data collection for the Smart-seq2 dataset: BCL files were demultiplexed with the bcl2fastq software from Illumina. After quality-control with FastQC, reads were aligned using rnaSTAR to the GRCm38 (mm10) genome with ERCC synthetic RNA added. Read counts were collected using the parameter “quantMode GeneCounts” of rnaSTAR and using the unstranded values.

Data analysis

1. Confocal images: all the image analysis and image processing were done using Imaris (64x version 9.2.0) and ImageJ 1.41 or Fiji image processing softwares.

2. Single-cell RNA-seq data analysis software and packages: BiocManager: 1.30.10, Cell Ranger: 3.0.2 (wild-type aging), 4.0.0 (Rag1KO 1st Batch) and 6.1.2 (Rag1KO 2nd Batch), Seurat: 3.2.3, SeuratObject: 4.0.2, gplots: 3.1.1, ggplot2: 3.3.2, magrittr: 2.0.2, reticulate: 1.16, RColorBrewer: 1.1-2, tidyverse: 1.3.0, scCODA: 0.1.6, pandas: 1.3.5, tensorflow: 2.5.2, sci-kit learn: 1.0.2, matplotlib: 3.5.1, seaborn: 0.11.2, Metascape: 3.5, DAVID 2021, STRING: 11.0-11.5

4. Statistical analyses were done using GraphPad Prism (GraphPad Software, Inc.).

For manuscripts utilizing custom algorithms or software that are central to the research but not yet described in published literature, software must be made available to editors and reviewers. We strongly encourage code deposition in a community repository (e.g. GitHub). See the Nature Portfolio guidelines for submitting code & software for further information.

Data

Policy information about availability of data

All manuscripts must include a data availability statement. This statement should provide the following information, where applicable:

- Accession codes, unique identifiers, or web links for publicly available datasets
- A description of any restrictions on data availability
- For clinical datasets or third party data, please ensure that the statement adheres to our policy

The datasets we use (scRNA-seq) are deposited at GEO (NCBI) under accession number GSE202579. Datasets re-analyzed in this study include data from GSE166548, GSE138891 and GSE132042. The rest of the data included in this study are available within the paper as data source files in the supplementary information.

Field-specific reporting

Please select the one below that is the best fit for your research. If you are not sure, read the appropriate sections before making your selection.

☒ Life sciences ☐ Behavioural & social sciences ☐ Ecological, evolutionary & environmental sciences

For a reference copy of the document with all sections, see nature.com/documents/nr-reporting-summary-flat.pdf

Life sciences study design

All studies must disclose on these points even when the disclosure is negative.

Sample size	No power analyses were used to predetermine sample sizes. However, sample sizes were chosen based on prior literature using similar experimental paradigms (for example doi:10.1016/j.celrep.2019.03.099; doi:10.1016/j.immuni.2017.08.008; doi:10.1016/j.cell.2017.05.018; doi:10.1016/j.neuron.2021.01.027
Data exclusions	No data were excluded from analysis.
Replication	For all mouse experiments, 3-6 mice per genotype were analyzed. For histological analysis, 3-6 random region of interest (ROIs) per each brain section were taken and three random brain sections per animal were quantified to account for variability within the biological sample. Replicaion was succesful for all conditions reported.
Randomization	The allocation of samples including brain sections was random.
Blinding	All data acquisition and analysis fwere done in a blinded manner. The experimentor was unblinded when preparing samples from IFNg because of the obvious differences in the phenotype.

Reporting for specific materials, systems and methods

We require information from authors about some types of materials, experimental systems and methods used in many studies. Here, indicate whether each material, system or method listed is relevant to your study. If you are not sure if a list item applies to your research, read the appropriate section before selecting a response.

Materials & experimental systems

n/a	Involved in the study	
☐	☒	Antibodies
☒	☐	Eukaryotic cell lines
☒	☐	Palaeontology and archaeology
☐	☒	Animals and other organisms
☒	☐	Human research participants
☒	☐	Clinical data
☒	☐	Dual use research of concern

Methods

n/a	Involved in the study	
☒	☐	ChIP-seq
☐	☒	Flow cytometry
☒	☐	MRI-based neuroimaging

Antibodies

Antibodies used	1.mouse anti APC (1:100) Millipore cat OP80-100UG<br>2.rabbit anti B2m (1:100) Abcam cat ab75853<br>3.rabbit anti STAT1 (1:250) Cell Signalling cat 14994S<br>4.rat anti CD8a (1:100) BioLegend cat 100702<br>5.rabbit anti Iba1 (1:500) Wako cat 234 004<br>6.goat anti Serpina3n (1:100) Bio-techne cat AF4709

7.rat anti C4b (1:25) Thermo fisher scientific cat MA1-40047
8.rabbit anti Olig2 (1:250) Millipore cat AB9610
9.mouse anti-Gstp (1:250) BD cat 610719
10.rabbit anti CD3e (1:200) Novus Biologicals cat NB600-1441
11.rat anti MHC class I (ER-HR52) (1:100) Santa Cruz cat sc-59199
12.goat anti-Rabbit IgG Antibody (H+L) Biotinylated (1:200) Vector cat BA-1000-1.5
13. Mouse anti galactocerebroside (O1) (1:5) raised in mouse hybridoma.
14.rat anti Mac2 (1:250) Biolegend cat 125402
15.chicken anti MBP (1:1000) thermo fisher scientific cat PA1-10008
16.chicken anti-Neurofilament heavy polypeptide(1:400) abcam cat ab4680
17. goat anti PDGFR alpha (1:100) R&D Systems cat AF1062
18. goat anti CD69 (1:100) R&D Systems cat AF2386
19. goat anti PD-1 (1:100) R&D Systems cat AF1021
20. rabbit anti-LAG-3 antibody (1:100) Abcam cat ab209238
21.CD16/CD32 Monoclonal Antibody (1:100) eBioscience cat 14-0161-82
22.CD11b (PE/Cy7, M1/70, 1:200) eBioscience Cat:48-0451-82

anti mouse 555 thermo fisher scientific (1:500) cat A-21422
anti mouse 647 thermo fisher scientific (1:500) cat A-21235
anti mouse 488 thermo fisher scientific (1:500) cat A-21202
anti rabbit 555 thermo fisher scientific (1:500) cat A-21428
anti rabbit 488 thermo fisher scientific (1:500) cat A-11008
anti rat 555 thermo fisher scientific (1:500) cat A-21434
anti goat 555 thermo fisher scientific (1:500) cat A-32116
anti rat 488 thermo fisher scientific (1:500) cat A-11006
anti chicken 488 thermo fisher scientific (1:500) cat A-11039
anti chicken 555 thermo fisher scientific (1:500)cat A-32932
Streptavidin, Alexa Fluor ™ 555 conjugate thermo fisher scientific (1:500) cat S32355
AF1062FM green fluorescent myelin stain (thermo fisher scientific F34651,1:400)
Donkey anti-Rat 488 thermo fisher scientific (1:500) cat A-21432
Donkey anti-Goat 555 thermo fisher scientific (1:500) cat A32816
Donkey anti-Rabbit 647 thermo fisher scientific (1:500) cat A-31573

Rat anti PD-1 bxcell cat BE0146
Rat isotype control bxcell cat BE0089
Hamster anti CTLA-4 bxcell cat BP0131
Hamster isotype control bxcell cat BP0087

Validation

All the primary antibodies were validated by extensive previous studies and by the manufacturers.
1. https://www.merckmillipore.com/DE/de/product/Anti-APC-Ab-7-Mouse-mAb-CC-1,EMD_BIO-OP80?ReferrerURL=https%3A%2F%2Fwww.google.com%2F
2.https://www.abcam.com/beta-2-microglobulin-antibody-ep2978y-ab75853.html
3. https://www.cellsignal.com/products/primary-antibodies/stat1-d1k9y-rabbit-mab/14994?site-search-type=Products&N=4294956287&Ntt=+14994s&fromPage=plp&_requestid=2125717
4.https://www.biolegend.com/en-ie/products/purified-anti-mouse-cd8a-antibody-157
5. https://labchem-wako.fujifilm.com/europe/product/detail/W01W0101-1974.html
6. https://www.rndsystems.com/products/mouse-serpin-a3n-antibody_af4709
7. https://www.thermofisher.com/antibody/product/Complement-C4-Antibody-clone-16D2-Monoclonal/MA1-40047
8. https://www.merckmillipore.com/DE/de/product/Anti-Olig-2-Antibody,MM_NF-AB9610?ReferrerURL=https%3A%2F%2Fwww.google.com%2F
9. https://www.bdbiosciences.com/en-us/products/reagents/microscopy-imaging-reagents/immunofluorescence-reagents/purified-mouse-anti-gst.610719
10.https://www.novusbio.com/products/cd3-antibody-sp7_nb600-1441
11.https://www.scbt.com/p/mhc-class-i-antibody-er-hr52?requestFrom=search
12.https://vectorlabs.com/biotinylated-goat-anti-rabbit-igg-antibody.html
14. https://www.biolegend.com/en-us/products/purified-anti-mouse-human-mac-2-galectin-3-antibody-4935
15.https://www.thermofisher.com/antibody/product/MBP-Antibody-Polyclonal/PA1-10008
16.https://www.abcam.com/neurofilament-heavy-polypeptide-antibody-ab4680.html
17.https://www.rndsystems.com/products/mouse-pdgf-ralpha-antibody_af1062
18.https://www.rndsystems.com/products/mouse-cd69-antibody_af2386
19.https://www.rndsystems.com/products/mouse-pd-1-antibody_af1021
20.https://www.abcam.com/lag-3-antibody-epr20294-77-ab209238.html
21.https://www.thermofisher.com/antibody/product/CD16-CD32-Antibody-clone-93-Monoclonal/14-0161-82
22.https://www.thermofisher.com/antibody/product/CD11b-Antibody-clone-M1-70-Monoclonal/25-0112-82

Secondary

https://www.thermofisher.com/antibody/product/Goat-anti-Mouse-IgG-H-L-Cross-Adsorbed-Secondary-Antibody-Polyclonal/A-21422
https://www.thermofisher.com/antibody/product/Goat-anti-Mouse-IgG-H-L-Cross-Adsorbed-Secondary-Antibody-Polyclonal/A-21235
https://www.thermofisher.com/antibody/product/Donkey-anti-Mouse-IgG-H-L-Highly-Cross-Adsorbed-Secondary-Antibody-

Polyclonal/A-21202
https://www.thermofisher.com/antibody/secondary/query/goat+anti-rabbit/filter/species/Rabbit?gclid=EAIaIQobChMIzYLDl8mL9AIVzEaRBR1esQ3fEAAYASAAEgKj4PD_BwE&ef_id=EAIaIQobChMIzYLDl8mL9AIVzEaRBR1esQ3fEAAYASAAEgKj4PD_BwE:G:s&s_kwcid=AL!3652!3!393949267183!b!!g!!%2Bthermo%20%2Banti%20%2Brabbit&cid=bid_pca_aus_r01_co_cp1359_pjt0000_bid00000_0se_gaw_bt_pur_con
https://www.thermofisher.com/antibody/product/Goat-anti-Rabbit-IgG-H-L-Cross-Adsorbed-Secondary-Antibody-Polyclonal/A-11008
https://www.thermofisher.com/antibody/product/Goat-anti-Rat-IgG-H-L-Cross-Adsorbed-Secondary-Antibody-Polyclonal/A-21434
https://www.thermofisher.com/antibody/product/Rabbit-anti-Goat-IgG-H-L-Cross-Adsorbed-Secondary-Antibody-Polyclonal/A-21431
https://www.thermofisher.com/antibody/product/Goat-anti-Rat-IgG-H-L-Cross-Adsorbed-Secondary-Antibody-Polyclonal/A-11006
https://www.thermofisher.com/order/catalog/product/S21381#/S21381
https://www.thermofisher.com/antibody/product/Goat-anti-Chicken-IgY-H-L-Secondary-Antibody-Polyclonal/A-11039
https://www.thermofisher.com/antibody/product/Goat-anti-Chicken-IgY-H-L-Cross-Adsorbed-Secondary-Antibody-Polyclonal/A32932
https://www.thermofisher.com/antibody/product/Donkey-anti-Goat-IgG-H-L-Cross-Adsorbed-Secondary-Antibody-Polyclonal/A-21432
https://www.thermofisher.com/antibody/product/Donkey-anti-Rat-IgG-H-L-Highly-Cross-Adsorbed-Secondary-Antibody-Polyclonal/A-21208
https://www.thermofisher.com/antibody/product/Donkey-anti-Rabbit-IgG-H-L-Highly-Cross-Adsorbed-Secondary-Antibody-Polyclonal/A-31573

in vivo treatment

https://bxcell.com/product/invivomab-anti-m-pd-1/
https://bxcell.com/product/rat-igg2a-isotype-control/
https://bxcell.com/product/m-cd152-m-ctla-4-2/
https://bxcell.com/product/invivoplus-polyclonal-syrian-hamster-igg/

Animals and other organisms

Policy information about studies involving animals; ARRIVE guidelines recommended for reporting animal research

Laboratory animals

Mouse: C57BL/6J Janvier Labs, CSD animal facility, University Hospital animal facility. Age 3,12,18,24 months,male
Mouse: Trem2 KO Prof. Christian Haass (Laboratory of Neurodegenerative Disease Research, DZNE, Munich), Turnbull et al., 2006. Age 18 months,male
Mouse : RAG1 KO (B6.129S7-Rag1tm1Mom/J), Prof. Arthur Liesz, laboratory of stroke immunology of Insitute of Stroke and dementia, LMU, Munich. Age 24 months ,male
Mouse : CD8 KO mice (B6.129S2-Cd8atm1Mak/J) Prof. Rudolf Martini, University Hospital Würzburg, Germany. Age 24 months old,male

Wild animals

No wild animals were used

Field-collected samples

No field collection samples were used in the study

Ethics oversight

All animal experiments performed in this work were in agreement with the German animal welfare law and state specific regulations for animal experiments

Note that full information on the approval of the study protocol must also be provided in the manuscript.

Flow Cytometry

Plots

Confirm that:

- [x] The axis labels state the marker and fluorochrome used (e.g. CD4-FITC).
- [x] The axis scales are clearly visible. Include numbers along axes only for bottom left plot of group (a 'group' is an analysis of identical markers).
- [x] All plots are contour plots with outliers or pseudocolor plots.
- [ ] A numerical value for number of cells or percentage (with statistics) is provided.

Methodology

Sample preparation

Four young (3-month-old), and four old (24-month-old) male C57BL/6 mice were deeply anesthetized and perfused with cold PBS (Sigma, D8537). Each brain was carefully removed and individually micro-dissected under a dissection microscope; grey matter was isolated from the frontal cortex and white matter was carefully isolated from the optic tract, medial lemniscus and corpus callosum (attached grey matter and choroid plexus were removed). We used our previous established isolation protocol37 using gentleMACS™ with the Neural Tissue Dissociation Kit (Papain) (Miltenyi Biotec) and a final concentration of 45 mM actinomycin D (Act-D, Sigma, No.A1410). Subsequently, cells were blocked with mouse FcR-blocking reagent (CD16/CD32 Monoclonal Antibody, eBioscience cat:14-0161-82, 1:100), stained with antibodies against CD11b (PE/Cy7, M1/70,

	eBioscience, Cat:48-0451-82,1:200) and afterwards washed with PBS. Before sorting, the cell suspensions were stained by the live/dead marker SYTOX Blue (Thermo Fisher, s34857, final concentration 1µM).
Instrument	Sony SH800S Cell Sorter
Software	The SH800S software for the data collection. FlowJo was used for the flow cytometry data analysis.
Cell population abundance	During single-cell library preparation we have confirmed the quality of the single-cells via qPCR assay (more details in methods section).
Gating strategy	CNS cells were gated for singlets by using FSC-A and FSC-H, followed by gating for living cell (SYTOX Blue negative population, fixable viability dye), then CD11b- cells were sorted.

☒ Tick this box to confirm that a figure exemplifying the gating strategy is provided in the Supplementary Information.
